# A mathematical analysis of rebound in a target-mediated drug disposition model: II. With feedback

**DOI:** 10.1007/s00285-016-1073-6

**Published:** 2016-11-10

**Authors:** Philip J. Aston, Gianne Derks, Balaji M. Agoram, Piet H. van der Graaf

**Affiliations:** 1 Department of Mathematics, University of Surrey, Guildford, GU2 7XH UK; 2 MedImmune, Pharmacokinetics/Dynamics and Bioanalysis, Cambridge, CB21 6GH UK; 3 Leiden Academic Centre for Drug Research (LACDR), 2300 RA Leiden, The Netherlands; 4 Certara QSP, Canterbury, CT2 7FG UK

**Keywords:** Systems pharmacology, Feedback, Antibody, Receptor rebound, Pharmacokinetics, Pharmacodynamics, 92C45, 92C50, 34E10, 37L25

## Abstract

We consider the possibility of free receptor (antigen/cytokine) levels rebounding to higher than the baseline level after the application of an antibody drug using a target-mediated drug disposition model. It is assumed that the receptor synthesis rate experiences homeostatic feedback from the receptor levels. It is shown for a very fast feedback response, that the occurrence of rebound is determined by the ratio of the elimination rates, in a very similar way as for no feedback. However, for a slow feedback response, there will always be rebound. This result is illustrated with an example involving the drug efalizumab for patients with psoriasis. It is shown that slow feedback can be a plausible explanation for the observed rebound in this example.

## Introduction

In this paper, we continue our investigation into the phenomenon of receptor rebound, i.e., a post-dose rise in receptor levels to higher than pre-dose (baseline). In our previous paper (Aston et al. 2014), we showed that if no homeostatic feedback is present, rebound will occur if and only if the elimination rate of the target-drug complex is slower than both the elimination rate of the drug and the elimination rate of the target. Binding to cell-surface receptors typically results in accelerated turnover of the anti-body and hence complex, so it can be expected that rebound will occur in rare cases only. However,  Ng et al. (2005) describe a treatment for psoriasis patients with efalizumab in which rebound is observed. They also derive a model that shows good agreement with the experimental observations. A main difference between this model and standard TMDD models is that the model includes receptor feedback for the synthesis rate. In the basic TMDD model, reduction of free target levels does not have any impact on the endogenous production or elimination rate of the free target, i.e., no endogenous feedback control exists to compensate for the antibody effect on target. The model in Ng et al. (2005) includes such feedback control via an additional differential equation for the synthesis rate. Another indicator of the importance of feedback in the synthesis rate is the recent paper by Kristensen et al. (2013), in which it is postulated that the protein synthesis rate is the predominant regulator of protein expression during differentiation.

In this paper we investigate how receptor feedback influences the occurrence of rebound in the TMDD model. The receptor feedback is usually a dynamical process via some moderators and we modify the TMDD model to include feedback by adding an additional differential equation. If the feedback is very fast, a quasi-equilibrium approach can be used and the feedback can be included in the synthesis term itself Hek (2010). This approach is similar to the one leading to the Michaelis–Menten approximation or the Quasi-Steady-State approximation. We will use the term “direct feedback” for the quasi-equilibrium approximation. The modification to include feedback is presented in full detail in Sect. 3, after a review of the main results of our first paper (Aston et al. 2014) on rebound in the basic TMDD model without feedback in Sect. 2. A wide class of feedback functions is considered with some natural assumptions on their action.

**Fig. 1:**
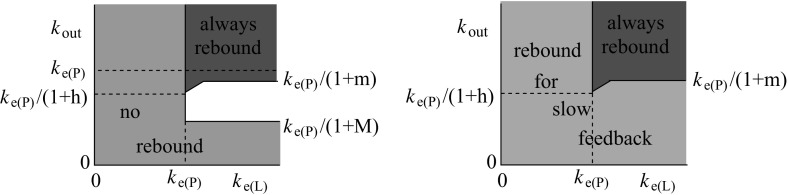
A schematic overview of the elimination plane. On the horizontal axis is the elimination rate of the ligand (antibody/drug), denoted by $k_\mathrm{e(L)}$, and on the vertical axis is the elimination rate of the receptor (antigen/target), denoted by $k_\mathrm{out}$. The elimination rate of the antibody–antigen complex (drug–target product) is denoted by $k_\mathrm{e(P)}$. On the *left* is an overview of the occurrence of rebound in the case of the direct feedback approximation. In the *red* region, rebound will occur and in the *green* region no rebound can occur. The symbols *h*, *m* and *M* are related to the feedback function and will be defined in Sect. 4. In the case where there is no feedback, $m=h=M=0$ and the region in which there is rebound is the *red* region above the line $k_\mathrm{out}=k_\mathrm{e(P)}$. On the *right* is an overview of the occurrence of feedback, depending on the speed of the feedback response. There is a *red* region in the elimination plane, that equals the direct feedback rebound region, for which rebound will occur for any speed of the feedback response. In addition, if the response is slow, rebound will occur for any elimination values. The precise response speed for which the rebound stops occurring will vary across the *pink* region (color figure online)

The TMDD model with the direct feedback approximation is analysed in Sect. 4. This analysis shows that the existence or non-existence of rebound is still linked to the elimination rates, though rebound can be expected in a larger region in the elimination parameter plane, see the left plot in Fig. 1. The general case of feedback via a moderator is analysed in Sect. 5. Now the response speed of the feedback moderator plays an important role as well as the elimination rates. It is shown that there is a similar region in the elimination rate plane as for the basic TMDD model for which rebound will occur for any response speed. Furthermore, if the feedback responds slowly to a change in the receptor levels, rebound will occur for any value of the elimination rates. A schematic overview of this result is in the right plot of Fig. 1.

The TMDD model is a one-compartment model. In Sect. 6, we consider briefly two classes of more general models with feedback (one class is related to multiple compartments, the other to more general feedback mechanisms) and show that rebound will generically occur in such models for slow feedback moderator response. In this paper, the word generic refers to the assumptions that the linearisation about the baseline state has no degenerate eigenvalues and that trajectories approaching the attracting baseline state do so tangent to the eigenvector associated with the least attracting eigenvalue. Section 7 illustrates the theory for two examples. The first example is a standard TMDD model for the IgE mAb omalizumab. We discuss what the effect of the two types of feedback mechanisms would be in this case . The second example is the model in Ng et al. (2005) which describes the efficacy of efalizumab for patients with psoriasis. Simulations show that the model does not lead to rebound if the feedback is turned off, but a significant rebound (about 140% of baseline) will occur if feedback is included. These results can be predicted with our analysis and underpin the observations of rebound in some patients. Finally, Sect. 8 contains a discussion of the results obtained in this paper and poses some open questions. The proofs of some of the technical results in the paper are given in the Appendix.

**Fig. 2:**
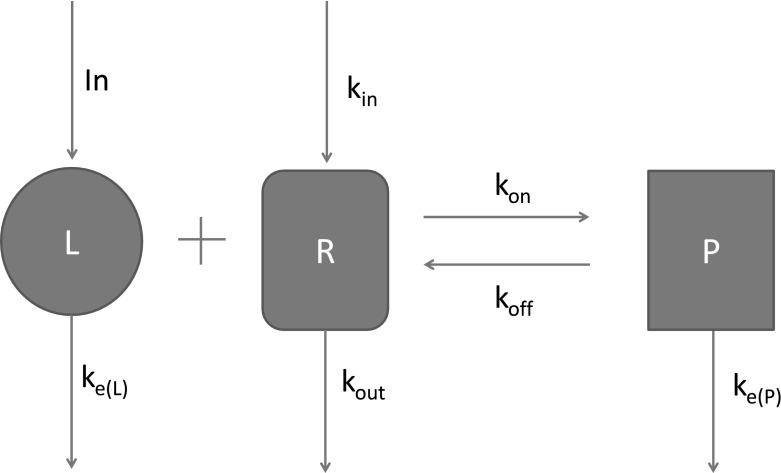
The TMDD reaction mechanism

## Review of previous results

In our previous paper (Aston et al. 2014), we considered a one-compartment TMDD model based on the original work of Levy (1994) where the ligand *L* binds reversibly with the receptor *R* to form a receptor-ligand complex *P* as shown in Fig. 2. The TMDD model assumes a mechanism-based reaction to explain the ligand-receptor interaction. The parameters of the model are the binding rate constants $k_\mathrm{on}$ and $k_\mathrm{off}$, the receptor turnover and elimination rates $k_\mathrm{in}$ and $k_\mathrm{out}$, and the elimination rates of the ligand and complex $k_\mathrm{e(L)}$ and $k_\mathrm{e(P)}$. The system is assumed to be initially at steady state, into which a single bolus infusion $L_0$ of the ligand into the central (plasma) compartment is made (represented in Fig. 2 by ‘In’). The differential equations that comprise the mathematical model for this system are given by

1 $$\begin{aligned} \frac{dL}{dt}= & {} -k_\mathrm{e(L)}L - k_\mathrm{on}LR + k_\mathrm{off}P \end{aligned}$$

2 $$\begin{aligned} \frac{dR}{dt}= & {} k_\mathrm{in}- k_\mathrm{out}R - k_\mathrm{on}LR + k_\mathrm{off}P \end{aligned}$$

3 $$\begin{aligned} \frac{dP}{dt}= & {} k_\mathrm{on}LR - k_\mathrm{off}P -k_\mathrm{e(P)}P \end{aligned}$$

A steady state of this system is given by $L=P=0$, $R=k_\mathrm{in}/k_\mathrm{out}$. Adding the bolus injection $L_0$ gives the initial conditions

4 $$\begin{aligned} L(0)=L_0, \quad R(0)=R_0=\frac{k_\mathrm{in}}{k_\mathrm{out}}, \quad P(0)=0. \end{aligned}$$

After the ligand is added to the system in its baseline state, initially the receptor level decreases, but after a while it goes up again and returns to its baseline value, since the steady state is globally asymptotically stable (Aston et al. 2014). Rebound occurs if, in the return to the baseline value, the receptor level increases to values above the baseline $R_0$. In Aston et al. (2014), we determined precise conditions for the existence and non-existence of rebound. Our main result was the following.

### Theorem 2.1

Rebound occurs in Eqs. (1)–(3) if and only if

$$\begin{aligned} k_\mathrm{e(P)}<k_\mathrm{e(L)}\quad \hbox {and}\quad k_\mathrm{e(P)}<k_\mathrm{out}. \end{aligned}$$

**Fig. 3:**
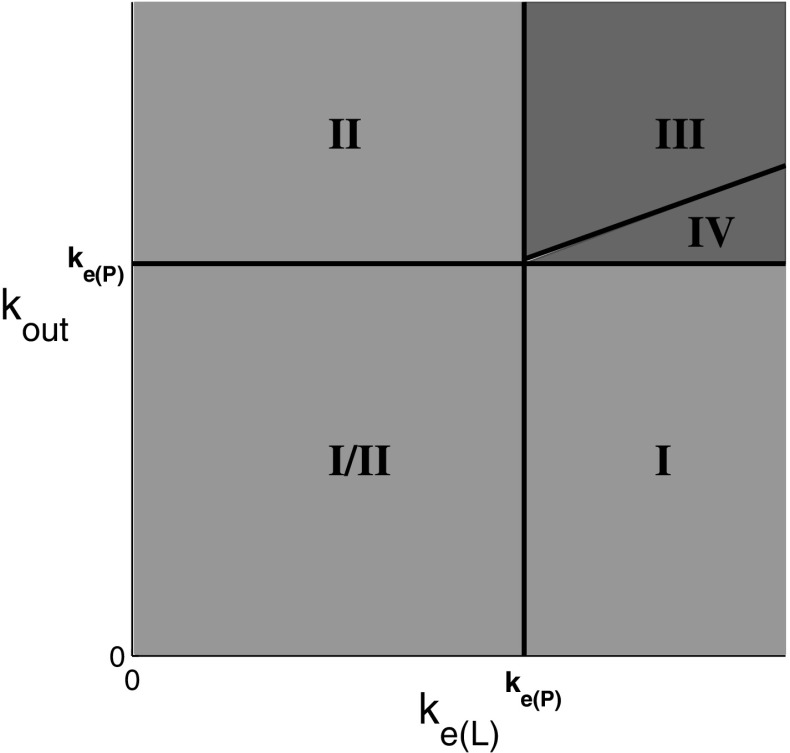
The *green* region shows the part of the $(k_\mathrm{e(L)},k_\mathrm{out})$ parameter plane where there is no rebound, and the *red* region shows where there is rebound. The boundaries are given by the lines $k_\mathrm{e(L)}=k_\mathrm{e(P)}$ and $k_\mathrm{out}=k_\mathrm{e(P)}$. This figure is similar to Fig. 2 in Aston et al. (2014) (color figure online)

This result shows that rebound occurs if and only if the elimination rate of the product is slower than the elimination rates of both the ligand and the receptor. This is represented graphically by different regions in the $(k_\mathrm{e(L)},k_\mathrm{out})$ plane, as shown in Fig. 3. The above condition for rebound in terms of non-dimensional parameters is given by

$$\begin{aligned} k_4<k_1\quad \mathrm{and}\quad k_4<k_3, \end{aligned}$$

where

$$\begin{aligned} k_1 = \frac{k_\mathrm{e(L)}}{k_\mathrm{on}R_0},\quad k_3=\frac{k_\mathrm{out}}{k_\mathrm{on}R_0}, \quad k_4=\frac{k_\mathrm{e(P)}}{k_\mathrm{on}R_0}. \end{aligned}$$

Intuitively, if the clearance of the complex is slow relative to the other two clearance rates, when there is a significant build-up of the complex due to the rapid binding of ligand and receptor, then this complex is only eliminated slowly. This results in an additional “production” of the receptor when the complex dissociates which is likely to lead to rebound. Our analysis quantifies this and we now show how these thresholds that we derived previously are affected if receptor feedback is added to the system.

## Including feedback in the TMDD model

We now extend our previous work on rebound in the basic TMDD model by extending the model to include feedback on the synthesis rate. The feedback gives an increase in the production rate of the receptor if the receptor levels drop under the baseline. Similarly, if the receptor level exceeds the baseline value, then the feedback induces a decrease in the production rate. Thus, this mechanism can be classified as negative feedback.

We start with the TMDD equations (1)–(3) but now assume that the rate of production of *R* ($k_\mathrm{in}$) is not constant but varies as *R* changes. Thus, Eq. (2) is now modified to have the form

5 $$\begin{aligned} \frac{dR}{dt} = k_\mathrm{in}F - k_\mathrm{out}R - k_\mathrm{on}LR + k_\mathrm{off}P. \end{aligned}$$

The new feedback variable *F*, which will be related to *R*, has the effect of varying the production rate of the receptor. The feedback is usually via some moderator and its dynamics is described with the differential equation

6 $$\begin{aligned} \frac{dF}{dt}=\alpha (H(R)-F), \quad F(0)=1. \end{aligned}$$

The constant $\alpha \ge 0$ determines the strength of the feedback and determines the time scale on which the feedback moderator responds to changes in the receptor levels. Clearly, if $\alpha =0$, then *F* is a constant and the model reduces to the basic TMDD model with no feedback. If $\alpha$ is small, the feedback response is slow, while if $\alpha$ is larger, there is a very fast feedback response. Formally, dividing (6) through by $\alpha$ and taking the limit $\alpha \rightarrow \infty$, we see that the derivative term vanishes and the fixed relation

7 $$\begin{aligned} F=H(R) \end{aligned}$$

is obtained. This motivates a quasi-equilibrium approximation in which the differential equation for *F* is replaced with the relation (7). We will call this the direct feedback approximation.

Next we consider the function *H*(*R*). The function must be such that the model has the following features:

- the production rate of the receptor, denoted by $k_\mathrm{in}F$, should be positive;
- the receptor concentration should have the baseline $R=R_0=k_\mathrm{in}/k_\mathrm{out}$;
- the feedback mechanism should reduce the production rate of the receptor when the receptor level exceeds the baseline and should increase it when the receptor level is below the baseline.

This leads to the following general assumptions on the function *H*(*R*):

1. $$H(R)>0$$ for all $$R\ge 0$$;
2. $$H(R_0)=1$$, where $$R_0=k_\mathrm{in}/k_\mathrm{out}$$;
3. $H(R)>1$ when $0\le R<R_0$ and $0<H(R)<1$ when $R>R_0$;
4. $$H\in C^2([0,\infty ))$$.

We note that assumption (H1) ensures that in case of the direct feedback approximation (i.e, *F* is a function of *R* and (7) holds) the feedback $F>0$, so that the production rate of *R* is always positive. Alternatively, if the dynamics of *F* is described by the differential equation (6), then this condition ensures that

$$\begin{aligned} \left. \frac{dF}{dt}\right| _{F=0}>0 \end{aligned}$$

and so the line $F=0$ cannot be crossed from above to below. This also ensures that $F>0$ for all $t>0$ and hence the production rate is always positive.

The basic TMMD model without feedback, Eqs. (1)–(3), has a steady state solution given by $L=P=0$, $R=R_0=k_\mathrm{in}/k_\mathrm{out}$. This will still be a steady state of our modified equations in both the full model and the direct feedback approximation, provided that (H2) holds. For the full model, we observe that the steady state value for *F* is $F=1$. Finally, the assumption (H3) ensures that the production of the receptor slows down when there is excess receptor, and increases when the receptor level is below the baseline and (H4) is a technical assumption assuring sufficient smoothness. Combining (H2)–(H4) implies that $H'(R_0)\le 0$. We note that if *H* is a positive, strictly decreasing, smooth function with $H(R_0)=1$, then (H1)–(H4) are satisfied. However, the stated conditions are more general and do not require *H* to be strictly decreasing.

We now have two modified TMDD models that incorporate feedback. For the direct feedback approximation, we use (7) to replace *F* with *H*(*R*) which gives the differential equations

8 $$\begin{aligned} \frac{dL}{dt}= & {} -k_\mathrm{e(L)}L - k_\mathrm{on}LR + k_\mathrm{off}P \end{aligned}$$

9 $$\begin{aligned} \frac{dP}{dt}= & {} k_\mathrm{on}LR - k_\mathrm{off}P -k_\mathrm{e(P)}P \end{aligned}$$

10 $$\begin{aligned} \frac{dR}{dt}= & {} k_\mathrm{in}H(R) - k_\mathrm{out}R - k_\mathrm{on}LR + k_\mathrm{off}P \end{aligned}$$

Alternatively, the full feedback model involves an extra differential equation for the moderator *F* and the modified TMDD model is given by

11 $$\begin{aligned} \frac{dL}{dt}= & {} -k_\mathrm{e(L)}L - k_\mathrm{on}LR + k_\mathrm{off}P \end{aligned}$$

12 $$\begin{aligned} \frac{dP}{dt}= & {} k_\mathrm{on}LR - k_\mathrm{off}P -k_\mathrm{e(P)}P \end{aligned}$$

13 $$\begin{aligned} \frac{dR}{dt}= & {} k_\mathrm{in}F - k_\mathrm{out}R - k_\mathrm{on}LR + k_\mathrm{off}P \end{aligned}$$

14 $$\begin{aligned} \frac{dF}{dt}= & {} \alpha (H(R)-F) \end{aligned}$$

The initial conditions for both models are given by (4), with the extra condition $F(0)=1$ for the feedback moderator.

We non-dimensionalise these equations in the same way as in Aston et al. (2014). We note that our new variable *F* is non-dimensional, and so we define the dimensionless variables

$$\begin{aligned} x= \frac{L}{L_0},\quad y= \frac{R}{R_0}, \quad z=\frac{P}{R_0}, \quad w=F, \quad \tau = k_\mathrm{on}R_0 t. \end{aligned}$$

We also define a new function

$$\begin{aligned} h(y)=H(R_0y). \end{aligned}$$

The above assumptions on the function *H*(*R*) translate into the following assumptions on *h*(*y*):

1. $$h(y)>0$$ for all $$y\ge 0$$;
2. $$h(1)=1$$;
3. $h(y)>1$ when $0\le y<1$ and $0<h(y)<1$ when $y>1$;
4. $$h\in C^2([0,\infty ))$$.

For later use, we define

15 $$\begin{aligned} h_0=-h'(1) \end{aligned}$$

and note that the assumptions (h2)–(h4) imply that $h_0\ge 0$.

In terms of the non-dimensional quantities, the direct feedback equations (8)–(10) become

16 $$\begin{aligned} \dot{x}= & {} -k_1 x - xy + \mu k_2 z \end{aligned}$$

17 $$\begin{aligned} \dot{z}= & {} \frac{xy}{\mu }- \left( k_2 + k_4\right) z \end{aligned}$$

18 $$\begin{aligned} \dot{y}= & {} k_3 (h(y) - y) - \frac{xy}{\mu } + k_2 z \end{aligned}$$

with initial conditions

19 $$\begin{aligned} x(0)=1,\quad z(0)=0,\quad y(0)=1, \end{aligned}$$

where dot denotes differentiation with respect to $\tau$ and the dimensionless parameters are defined as in Aston et al. (2014) by

$$\begin{aligned} \mu =\frac{R_0}{L_0}, \quad k_1 = \frac{k_\mathrm{e(L)}}{k_\mathrm{on}R_0}, \quad k_2= \frac{k_\mathrm{off}}{k_\mathrm{on}R_0}, \quad k_3=\frac{k_\mathrm{in}}{k_\mathrm{on}R_0^2}=\frac{k_\mathrm{out}}{k_\mathrm{on}R_0}, \quad k_4=\frac{k_\mathrm{e(P)}}{k_\mathrm{on}R_0}. \end{aligned}$$

Clearly, this choice of non-dimensionalisation requires that $k_\mathrm{on}\ne 0$ and $k_\mathrm{in}\ne 0$ (so that $R_0\ne 0$). We note that all parameters must be non-negative due to their physical meaning, and additionally we will assume that they are in fact all strictly positive. The three variables *x*, *y*, and *z* are related to physical quantities and so must also be non-negative.

Similarly, the non-dimensional equations for the full feedback model (11)–(14) are given by

20 $$\begin{aligned} \dot{x}= & {} -k_1 x - xy + \mu k_2 z \end{aligned}$$

21 $$\begin{aligned} \dot{z}= & {} \frac{xy}{\mu }- \left( k_2 + k_4\right) z \end{aligned}$$

22 $$\begin{aligned} \dot{y}= & {} k_3 (w - y) - \frac{xy}{\mu } + k_2 z \end{aligned}$$

23 $$\begin{aligned} \dot{w}= & {} \epsilon (h(y)-w) \end{aligned}$$

with initial conditions

24 $$\begin{aligned} x(0)=1,\quad z(0)=0,\quad y(0)=1,\quad w(0)=1. \end{aligned}$$

Furthermore,

25 $$\begin{aligned} \epsilon =\frac{\alpha }{k_\mathrm{on}R_0} \end{aligned}$$

is a non-dimensional measure for the response speed of the feedback moderator.

## Analysis of the TMDD model with direct feedback

In this section, we will analyse the TMDD model with the direct feedback approximation, i.e., the Eqs. (16)–(18), with initial conditions (19), and seek to determine conditions for the existence or non-existence of rebound in this model. However, we start by proving some basic results for the model equations.

### Invariance, steady states and stability

For the basic TMDD model, we proved in Aston et al. (2014) that the positive octant $x,y,z\ge 0$ is invariant; that there is a unique steady state in the positive octant; and that this steady state is a global attractor. In this section we show that the same properties hold for the TMDD model with direct feedback. The proofs of these results can be found in Appendix A. We start by stating invariance of the positive octant.

#### Lemma 4.1

The octant of ${\mathbb {R}}^3$ defined by $x,y,z\ge 0$ is invariant under the flow of Eqs. (16)–(18).

This result shows that the equations are a good model in the sense that the concentrations of the ligand, complex and receptor can never go negative. Next we consider the steady states of Eqs. (16)–(18).

#### Lemma 4.2

In the region of ${\mathbb {R}}^3$ defined by $x,y,z\ge 0$, the Eqs. (16)–(18) have a unique steady state given by

26 $$\begin{aligned} x=0,\quad y=1,\quad z=0. \end{aligned}$$

This steady state is globally asymptotically stable.

This result shows that for all non-negative initial conditions and for all positive parameter values, each of the variables will converge to their unique steady state value.

It is also straightforward to show that the eigenvalues of the Jacobian of Eqs. (16)–(18) evaluated at the steady state (26) are very similar to those given in Aston et al. (2014). In particular, the two eigenvalues $\lambda _1$ and $\lambda _2$ are the same as previously and are given by

27 $$\begin{aligned} \lambda _1= & {} \frac{1}{2}\left( -(1+k_1+k_2+k_4)+\sqrt{(1+k_1-k_2-k_4)^2+4k_2}\right) \end{aligned}$$

28 $$\begin{aligned} \lambda _2= & {} \frac{1}{2}\left( -(1+k_1+k_2+k_4)-\sqrt{(1+k_1-k_2-k_4)^2+4k_2}\right) \end{aligned}$$

while the third eigenvalue, which previously was $\lambda _3=-k_3$, is now given by

29 $$\begin{aligned} \lambda _3=-k_3(h_0+1), \end{aligned}$$

where we recall that $h_0= -h'(1)$. The corresponding eigenvectors are given by

30 $$\begin{aligned} v_i= & {} \begin{pmatrix} \mu (\lambda _i+k_3(1 +h_0))(k_2+k_4+ \lambda _i) \ \lambda _i+k_3(1 +h_0) (\lambda _i +k_4) \end{pmatrix}, \nonumber \\&\quad i=1,2; v_3 = \begin{pmatrix}0\\ 0\\ 1 \end{pmatrix}. \end{aligned}$$

### Receptor dynamics

Before considering the full model, we first consider the effect of feedback on the dynamics of *y*, the non-dimensional form of the receptor, in the absence of the ligand or product. As with the basic TMDD model, we note that $x=z=0$ satisfies (16) and (17). Substituting $x=z=0$ into (18), we obtain the single differential equation

31 $$\begin{aligned} \dot{y}=k_3(h(y)-y),\quad y(0)=y_0. \end{aligned}$$

Assumption (h2) ensures that the previous steady state value $y=1$ is also a steady state of this equation while assumption (h3) ensures that this is the only non-negative steady state solution and that $\dot{y}<0$ if $y>1$ and $\dot{y}>0$ if $0\le y<1$. The theory of scalar, autonomous differential equations (Jordan and Smith 2007) then tells us that if the initial condition $y_0$ is less than the steady state ($y_0<1$) then $y(\tau )$ will increase monotonically to the steady state, whereas if $y_0$ is greater than the steady state ($y_0>1$), then $y(\tau )$ will decrease monotonically to the steady state. As $y(\tau )$ is monotonic, there is no possibility of rebound occurring in the dynamics of the receptor on its own.

Assumptions (h2) and (h3) together with definition (15) imply that

$$\begin{aligned} h(y)-y = -(h_0+1)(y-1) + O((y-1)^2). \end{aligned}$$

As $h_0\ge 0$, the inclusion of the function *h*(*y*) ensures that the asymptotic rate of convergence to the steady state (proportional to $e^{-k_3(h_0+1)t}$) will be faster than or equal to the rate of convergence which occurs when $h(y)=1$ (proportional to $e^{-k_3 t}$). Thus, qualitatively, the direct feedback has the effect of speeding up the monotonic convergence to the steady state in the absence of ligand and product, but there is no rebound in this case, i.e., if $y_0<1$, then $y(\tau )< 1$ for all $\tau \ge 0$.

### Conditions for rebound

Having established that feedback does not lead to rebound in the absence of the ligand, we now investigate the effect of adding the ligand. A local approximation of the feedback near the baseline will give a linear feedback function. So we first consider the special case that the feedback function *h*(*y*) is linear for $y\le 1$. It turns out to be straightforward to extend our results for the TMDD model without feedback to this case. The general nonlinear case will be analysed next and builds on the ideas of the linear analysis.

#### A mainly linear feedback function

We consider a feedback function which is linear on a *y*-interval including [0, 1]:

32 $$\begin{aligned} h(y)=\left\{ \begin{array}{ll}1+h_0(1-y),&{}\quad 0\le y\le 1+\frac{\beta }{h_0}; \\ h_1(y),&{}\quad y> 1+\frac{\beta }{h_0} \end{array}\right. \end{aligned}$$

where $h_0>0$, $0<\beta < 1$, and $h_1\in C^2\left( \left( {1+\frac{\beta }{h_0}},\infty \right) \right)$, $h_1(y)\in (0,1)$, with

$$\begin{aligned} \lim _{y\downarrow 1+\frac{\beta }{h_0}} h_1(y) = 1 -\beta , \hbox { } \lim _{y\downarrow 1+\frac{\beta }{h_0}} h'_1(y) = -h_0,\hbox { and } \lim _{y\downarrow 1+\frac{\beta }{h_0}} h''_1(y) = 0. \end{aligned}$$

This function satisfies all the conditions (h1)–(h4). For the linear part of the function *h*, we note that $h(y)-y=(1+h_0)(1-y)$ and so Eq. (18) becomes

33 $$\begin{aligned} \dot{y}=\left\{ \begin{array}{ll} k_3(1+h_0)(1-y)-\frac{xy}{\mu }+k_2z, &{}\quad 0\le y\le 1+\frac{{{\beta }}}{h_0};\\ k_3({{h_1(y)}}-y)-\frac{xy}{\mu }+k_2z, &{}\quad y> 1+\frac{{{\beta }}}{h_0}. \end{array} \right. \end{aligned}$$

We then have the following result.

##### Theorem 4.3

Rebound occurs in Eqs. (16), (17), (33) if and only if

$$\begin{aligned} k_4<k_1\quad \hbox {and}\quad k_4<k_3(1+h_0). \end{aligned}$$

##### Proof

We note that for $0\le y\le 1$, Eqs. (16), (17) and (33) are the same as the basic TMDD model that we studied in Aston et al. (2014) except that $k_3$ has been replaced by $k_3(1+h_0)$. We claim that the results of Theorem 3.1 of Aston et al. (2014) apply in this case also after making this parameter replacement. To see this, we note that when proving the absence of rebound the only relevant values of *y* are $0\le y\le 1$ and so the matching point $y=1+\beta /h_0$ in (33) is not reached and the feedback function for $y>1$ is therefore not relevant. Moreover, the proof of rebound in Aston et al. (2014) involves only the asymptotic behaviour near to the globally stable steady state $(x,y,z)=(0,1,0)$ and the dynamics for $0\le y\le 1+\frac{{{\beta }}}{h_0}$ will give all the necessary information to determine this asymptotic behaviour. Hence, we can conclude that Theorem 3.1 in Aston et al. (2014) can be applied in this situation with $k_3$ replaced by $k_3(1+h_0)$. $\square$

Converting back to the dimensional parameters gives the following result.

##### Corollary 4.4

Consider the mainly linear feedback function

34 $$\begin{aligned} H(R)=\left\{ \begin{array}{ll}1+H_0(R_0-R),&{}\quad 0\le R\le R_0+\frac{{{\beta }}}{H_0}\\ H_1(R),&{}\quad R>R_0+\frac{{{\beta }}}{H_0}\end{array}\right. \end{aligned}$$

where $H_0>0$, $0<\beta <1$, $H_1(R)\in C^2((R_0+\frac{\beta }{H_0},\infty ))$, $H_1(R)\in (0,1)$ and $H_1$ and its first and second derivatives match the linear function at $R=R_0+\beta /H_0$.

Rebound occurs in the model given by Eqs. (8)–(10) with this feedback function if and only if

$$\begin{aligned} k_\mathrm{e(P)}<k_\mathrm{e(L)}\quad \hbox {and}\quad k_\mathrm{e(P)}<k_\mathrm{out}(1+R_0H_0). \end{aligned}$$

**Fig. 4:**
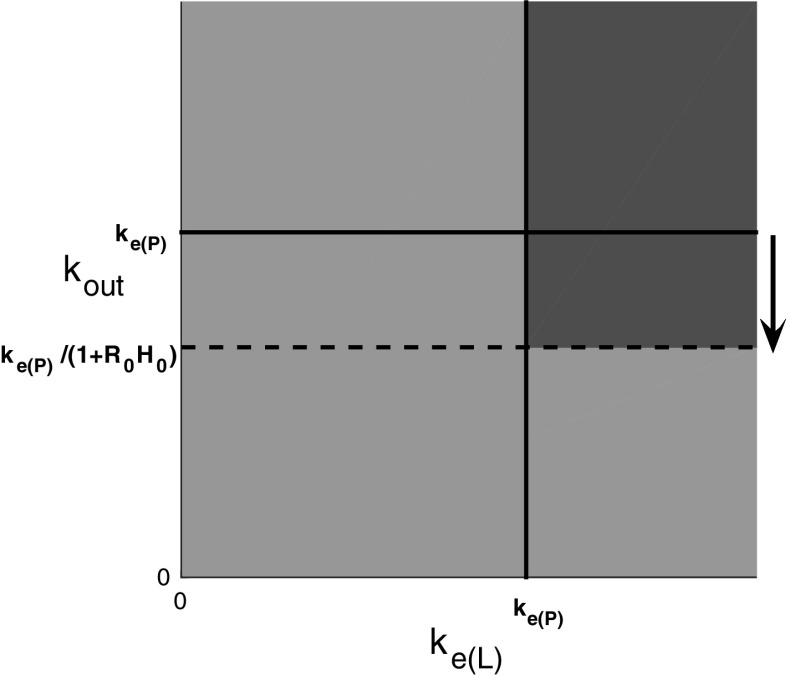
The *green* region shows the part of the $(k_\mathrm{e(L)},k_\mathrm{out})$ parameter plane where there is no rebound, and the *red* region shows where there is rebound for *H*(*R*) given by (34). The *solid line* $k_\mathrm{out}=k_\mathrm{e(P)}$ denotes the boundary of the rebound region when no feedback is present and the *dashed line* $k_\mathrm{out}=k_\mathrm{e(P)}/(1+R_0H_0)$ denotes the boundary of the rebound region when feedback is present (color figure online)

The rebound region in this case is shown in Fig. 4. We note by comparison with Fig. 3 that this mainly linear feedback function has enlarged the region in which rebound occurs compared to the case with no feedback.

#### A general nonlinear feedback function

We now consider the more general case where the feedback function *h* is nonlinear. We express the function *h*(*y*) in the form

35 $$\begin{aligned} h(y)=1+(1-y)\tilde{h}(y). \end{aligned}$$

Differentiating (35), evaluating at $y=1$ and using (15), we see that

36 $$\begin{aligned} \tilde{h}(1)=h_0. \end{aligned}$$

Condition (h3) implies that

37 $$\begin{aligned} \tilde{h}(y)>0,\quad \mathrm{when}\quad 0\le y<1, {{\hbox { and } 0<\tilde{h}(y)}}<\frac{1}{y-1}, \hbox { when } y>1. \end{aligned}$$

With this form of *h*, Eq. (18) becomes

38 $$\begin{aligned} \dot{y}=k_3(1-y)(1+\tilde{h}(y))-\frac{xy}{\mu }+k_2z \end{aligned}$$

##### Remark

We note that expanding *h*(*y*) in a Taylor series with remainder gives that $\tilde{h}(y)=-h'(\xi (y))$ where $\xi (y)$ lies between *y* and 1. However, the fact that $\xi (y)$ is an unknown function is not helpful, which is why we use the form of *h* given in (35).

We now consider regions similar to the ones defined for the model without feedback in Aston et al. (2014). First we consider region II and show that no rebound occurs in this region.

##### Theorem 4.5

Rebound does not occur if $k_1\le k_4$.

##### Proof

Following through the proof of Theorem 3.4 of Aston et al. (2014) for these modified equations, the only difference is that

$$\begin{aligned} \dot{y}|_{y=0}=k_3(1+\tilde{h}(0))+k_2z. \end{aligned}$$

Now $\tilde{h}(0)>0$ by (37) and so $\dot{y}|_{y=0}>0$ when $z\ge 0$, which is the condition required on this plane in the proof of Theorem 3.4 of Aston et al. (2014). Thus, the result holds in this case also. $\square$

The next result considers a region similar to region III and we show that rebound does occur.

##### Theorem 4.6

Rebound occurs if $k_1>k_4$ and $k_3(1+h_0)>-\lambda _1(k_1,k_2,k_4)$, where $\lambda _1$ is given by (27).

##### Proof

The Jacobian matrix for Eqs. (16), (17) and (38) evaluated at the steady state is the same as that for the simple TMDD model given in Aston et al. (2014) but with $k_3$ replaced by $k_3(1+h_0)$. The proof of Theorem 3.5 of Aston et al. (2014) (region III) is entirely based on the eigenvalues and eigenvectors of this matrix, and so the result holds in this case also, with the appropriate replacement of $k_3$. Since $\lambda _1$ does not involve $k_3$, there is no change to this eigenvalue. $\square$

The proofs in regions I and IV of the $(k_1,k_3)$ parameter plane in Aston et al. (2014) both made use of the fact that for the model without feedback the plane $v=y+z=1$ is invariant when $k_3=k_4$. However, this is no longer the case for our modified equations which include feedback. The differential equation for the dimensionless variable associated with the total amount of receptor $v=y+z$ is now given by

39 $$\begin{aligned} \dot{v}= (1-y)\left( k_3(1+\tilde{h}(y))-k_4\right) -k_4(v-1) \end{aligned}$$

and so

40 $$\begin{aligned} \dot{v}|_{v=1}=(1-y)\left( k_3(1+\tilde{h}(y))-k_4\right) . \end{aligned}$$

Clearly no condition on the parameters will give this derivative to be zero (unless $\tilde{h}$ is constant, the case considered in the previous section), and so we see, as already stated, that the plane $v=1$ is no longer invariant. However, the function *h*(*y*) has a continuous derivative by (h4) and this implies that $\tilde{h}(y)$ is continuous. Thus the Extreme Value Theorem for continuous functions gives that $\tilde{h}(y)$ has a minimum and a maximum for $y\in [0,1]$. So we define

41 $$\begin{aligned} m=\min _{y\in [0,1]}\tilde{h}(y),\quad M=\max _{y\in [0,1]}\tilde{h}(y). \end{aligned}$$

Using (36), this immediately implies that

42 $$\begin{aligned} m\le h_0\le M. \end{aligned}$$

Also, since $\tilde{h}(y)>0$ for all $0\le y<1$ by (37), we get

43 $$\begin{aligned} m\ge 0. \end{aligned}$$

Since *m* and *M* are the minimum and maximum of $\tilde{h}(y)$, then clearly

$$\begin{aligned} m\le \tilde{h}(y)\le M\quad \hbox {for all }\; 0\le y\le 1, \end{aligned}$$

and substituting for $\tilde{h}(y)$ from (35) and rearranging gives

44 $$\begin{aligned} 1+m(1-y)\le h(y)\le 1+M(1-y)\quad \hbox {for all }\; 0\le y\le 1. \end{aligned}$$

**Fig. 5:**
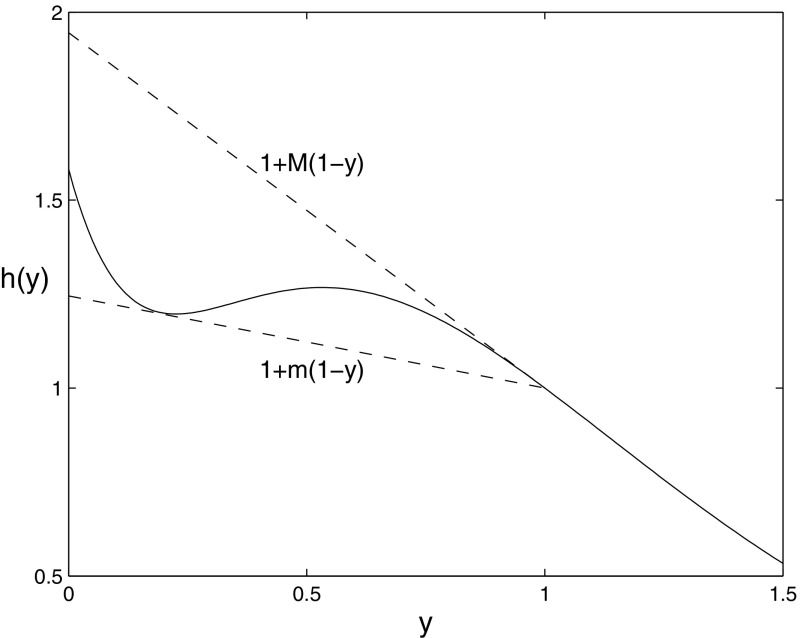
The function *h*(*y*) (*solid curve*) is bounded by two straight lines (*dashed lines*) with slopes $-m$ and $-M$ for $y\in [0,1]$

Graphically, this means that on the interval $y\in [0,1]$, the function *h*(*y*) is bounded by two straight lines, both of which pass through the point (1, 1), and which have slopes $-m$ and $-M$. This situation is illustrated in Fig. 5. Note that the Mean Value Theorem gives that $-M\ge \min \{h'(y)\mid 0\le y \le 1\}$ and $-m \le \max \{h'(y)\mid 0\le y \le 1\}$.

With the bounds *m* and *M*, we can give a slightly weaker version of Lemma 2.3 in Aston et al. (2014) and get regions in the $(k_3,k_4)$-plane for which the dimensionless variable *v* associated with the total amount of receptor stays on one side of the plane $v=1$.

##### Lemma 4.7

For all $\tau \ge 0$, the dimensionless variable *v* associated with the total amount of receptor satisfies

$$\begin{aligned} v(\tau ) \le 1,&\quad \hbox {if}&k_4 \ge k_3(1+M);\\ v(\tau ) \ge 1,&\quad \hbox {if}&k_4 \le k_3(1+m). \end{aligned}$$

##### Proof

We consider the vector field at the plane $v=1$. Equation (40) gives

$$\begin{aligned} \dot{v}|_{v=1}=(1-y)\left( k_3(1+\tilde{h}(y))-k_4\right) . \end{aligned}$$

Since we have restricted attention to the plane $v=1$, this implies that $y+z=1$ and so $y\le 1$ as *z* must be non-negative. As $1-y$ is non-negative, we can use the bounds on $\tilde{h}(y)$ given in (41) to get

45 $$\begin{aligned} (1-y)\left( k_3(1+m)-k_4\right) \le \dot{v}|_{v=1}\le (1-y)\left( k_3(1+M)-k_4\right) \end{aligned}$$

First we consider the case $k_4 \ge k_3(1+M)$. In this case, (45) gives $\dot{v}|_{v=1}\le (1-y)\left( k_3(1+M)-k_4\right) \le 0$, hence the vector field on the plane $v=1$ is either tangent to $v=1$ or points towards $v<1$. This implies that the region of phase space defined by $x,y,z\ge 0$, $v=y+z\le 1$ is invariant in forward time since the vector field on the four boundary planes is either tangent to the plane or points into the region (Smith 1995). This was proved for the planes $x=0$, $y=0$ and $z=0$ in Lemma 4.1 and for the plane $v=1$ above. Since $v(0)=1$ and this region is invariant, we conclude that $v(\tau )\le 1$ for all $\tau \ge 0$.

Next we consider the case $k_4 \le k_3(1+m)$. Now the other inequality in (45) gives $\dot{v}|_{v=1}\ge (1-y)\left( k_3(1+m)-k_4\right) \ge 0$, hence the vector field on the plane $v=1$ is either tangent to $v=1$ or points towards $v>1$. This implies that the region of phase space defined by $x,y,z\ge 0$, $v=y+z\ge 1$ is invariant in forward time. Since $v(0)=1$, this implies that $v(\tau )\ge 1$ for all $\tau \ge 0$. $\square$

With this observation, we can now derive a result on rebound for regions similar to regions I and IV in Aston et al. (2014).

##### Theorem 4.8

Define *m* and *M* as in (41), i.e., the function *h*(*y*) satisfies (44).

(i) Rebound does not occur if
  46 $$\begin{aligned} k_3\le \frac{k_4}{1+M} \end{aligned}$$
(ii) Rebound does occur if
  47 $$\begin{aligned} \frac{k_4}{1+m}<k_3<-\frac{\lambda _1}{1+h_0} \end{aligned}$$

Using (42) and (43), we note that

$$\begin{aligned} 0<\frac{k_4}{1+M}\le \frac{k_4}{1+h_0}\le \frac{k_4}{1+m}\le k_4. \end{aligned}$$

This illustrates in particular that there is no overlap between the region for which we have shown the existence of rebound and the region for which we have shown that there is no rebound.

##### Proof of Theorem 4.8

(i) The case when (46) holds is similar to region I in Aston et al. (2014) and has the property that $v(\tau )\le 1$ for all $\tau \ge 0$ by Lemma 4.7. Thus for all $\tau \ge 0$, we have $y(\tau )\le v(\tau )\le 1$ and so rebound cannot occur in this case.
(ii) When (47) holds (a condition similar to the one for region IV in Aston et al. (2014)), Lemma 4.7 ensures that $v(\tau )\ge 1$ for all $\tau \ge 0$. The right hand inequality in (47) implies that $\lambda _1<\lambda _3=-k_3(1+h_0)$ and so the eigenvalue closest to zero is $\lambda _3$, as occurred in region IV previously. Thus, trajectories will generically approach the steady state tangent to the *y*-axis, since the eigenvector associated with the eigenvalue $\lambda _3$ points along this axis. As the trajectory always satisfies $v\ge 1$ for all $\tau \ge 0$ then we again conclude that generically, almost all orbits will approach $y=1$ from above and hence rebound occurs.
  We must again eliminate the possibility that an orbit might approach the steady state tangent to one of the other eigenvectors for a particular choice of parameters. As previously, the linearised manifold in the direction of the eigenvector $v_2$ is outside of the phase space and so an orbit cannot approach tangent to this one-dimensional manifold.
  The one-dimensional linearised manifold in the direction of the eigenvector $v_1$ given in (30) is
  $$\begin{aligned} \left[ \begin{array}{c} x\\ z\\ y \end{array}\right] = \left[ \begin{array}{c} 0\\ 0\\ 1\end{array}\right] +a\, \left[ \begin{array}{c} \mu (k_2+k_4+\lambda _1)(\lambda _1+k_3(1+h_0))\\ ~~\,\lambda _1+k_3(1+h_0)\\ -(\lambda _1+k_4)\end{array}\right] . \end{aligned}$$
  We note that $\lambda _1+k_3(1+h_0)<0$ from the right hand inequality in (47). Thus, we must take $a<0$ to ensure that this linearised manifold gives positive values of *x* and *z* (since $k_2+k_4+\lambda _1>0$ by Lemma 2.7 of Aston et al. 2014). The linearised manifold for $v=y+z$ is then given by
  $$\begin{aligned} v=1+a\,(k_3(1+h_0)-k_4). \end{aligned}$$
  Using (42) and the left hand inequality of (47) gives $k_3(1+h_0)-k_4\ge k_3(1+m)-k_4>0$. Thus, this one-dimensional linearised manifold has a negative slope and so occurs for $v<1$. Since the trajectory in this case must satisfy $v\ge 1$, it is clearly not possible for it to approach the steady state tangent to this manifold. Thus, we conclude that all trajectories must approach the steady state tangent to the *y*-axis from above and hence rebound will occur. $\square$

In Theorems 4.6 and 4.8, we have proved that rebound occurs on both sides of the line $\lambda _1=\lambda _3$ with $k_3>k_4/(1+m)$. We now show that there is also rebound along this line.

##### Theorem 4.9

Rebound occurs if $k_3=-\lambda _1/(1+h_0)$ and $k_3>k_4/(1+m)$.

##### Proof

The proof uses exactly the same arguments as the proof of Theorem 3.7 in Aston et al. (2014). Along the curve $k_3=-\lambda _1/(1+h_0)$, the eigenvalues $\lambda _1$ and $\lambda _3$ collide and have one eigenvector, which is along the *y*-axis, and one generalised eigenvector. Thus all solutions will asymptotically align with the *y*-axis. Since $k_4/(1+m)<k_3$, Lemma 4.7 gives that $v(\tau )\ge 1$ and the intersection of the *y*-axis and the region $v(\tau )\ge 1$ is the part of the *y*-axis with $y\ge 1$. Hence all orbits must approach the steady state $(x,y,z)=(0,1,0)$ from above and rebound will occur. $\square$

We summarise the results of Theorems 4.5, 4.6, 4.8 and 4.9 as follows.

##### Theorem 4.10

Define

48 $$\begin{aligned} m = \inf \left\{ \left. \frac{h(y)-1}{1-y}\,\right| \, 0\le y< 1\right\} \quad \hbox {and} \quad M = \sup \left\{ \left. \frac{h(y)-1}{1-y}\,\right| \, 0\le y < 1\right\} . \end{aligned}$$

For the model equations (16)–(18) and for any function *h*(*y*) satisfying the assumptions (h1)–(h4)

- there is no rebound if $k_1\le k_4$ or $k_1>k_4$ and $k_3\le k_4/(1+M)$;
- rebound does occur if $k_1>k_4$ and $k_3>\min \left( k_4/(1+m), -\lambda _1/(1+h_0)\right)$.

We can also express these results in the terms of the dimensional parameters. Let *H*(*R*) be a function satisfying the assumptions (H1)–(H4) and define

$$\begin{aligned} m= & {} \inf \left\{ \left. \frac{(H(R)-1)R_0}{R_0-R}\,\right| \, 0\le R<R_0\right\} \quad \hbox {and}\quad M=\sup \left\{ \left. \frac{(H(R)-1)R_0}{R_0-R}\,\right| \,\right. \\&\left. 0\le R <R_0\right\} . \end{aligned}$$

For the model equations (8)–(10) with $H_0=-H'(R_0)$

- there is no rebound if $k_\mathrm{e(L)}\le k_\mathrm{e(P)}$ or $k_\mathrm{e(L)}>k_\mathrm{e(P)}$ and $k_\mathrm{out}<k_\mathrm{e(P)}/(1+M)$;
- rebound does occur if $k_\mathrm{e(L)}>k_\mathrm{e(P)}$ and $k_\mathrm{out}>\min \left( k_\mathrm{e(P)}/(1+m), -\lambda _1k_\mathrm{on}R_0/\right. \left. (1+R_0H_0)\right)$.

**Fig. 6:**
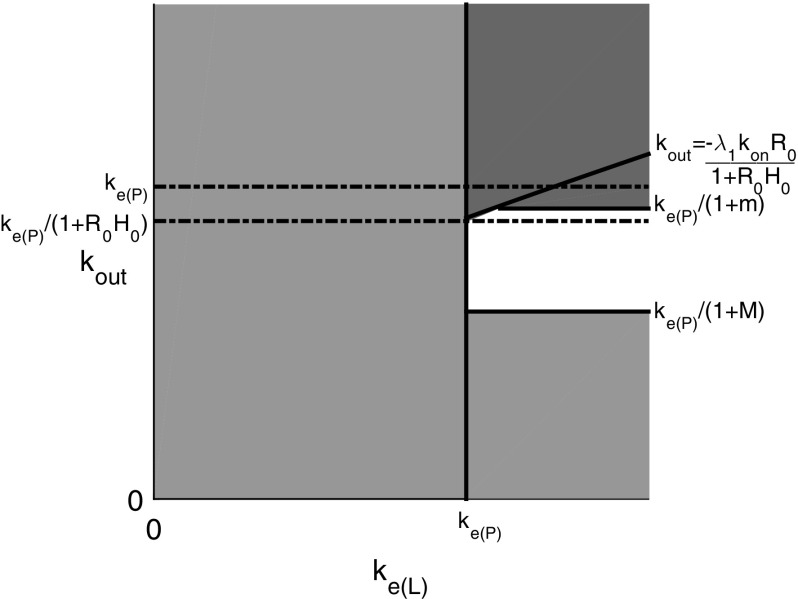
Regions in the $(k_\mathrm{e(L)},k_\mathrm{out})$ plane where rebound does and does not occur for the nonlinear feedback function *H*(*R*). *Red* indicates regions where rebound will occur and *green* indicates regions where rebound does not occur. In the *white* region, rebound may or may not occur depending on the particular feedback function *H* (color figure online)

These results are illustrated in Fig. 6.

We now focus on a few aspects of the theorem and compare it with the case where there is no feedback ($F=1$, and so $h_0=0=H_0$).

- In the equations without feedback, rebound occurred in the region $k_\mathrm{e(P)}<k_\mathrm{e(L)}$ and $k_\mathrm{e(P)}<k_\mathrm{out}$ (regions III and IV) and with feedback, rebound also occurs in this region.
- In the equations without feedback, there was no rebound in the region $k_\mathrm{e(L)}\le k_\mathrm{e(P)}$ (region II) and with feedback, there is also no rebound in this region.
- In the equations without feedback, there was no rebound in the region $k_\mathrm{out}<k_\mathrm{e(P)}<k_\mathrm{e(L)}$ (the part of region I that lies outside region II), but with feedback, there is rebound in some parts of this region but not in others, the precise details depend on the function *h*.
- In the region $k_\mathrm{e(L)}>k_\mathrm{e(P)}$, $k_\mathrm{e(P)}/(1+M)\le k_\mathrm{out}\le \min \left( k_\mathrm{e(P)}/(1+m), -\lambda _1/\right. \left. (1+h_0)\right)$, we have no general results, and the existence or otherwise of rebound will depend on the particular function *h*.
- If *H* is mainly linear, i.e. $F=H(R)=1+H_0(R_0-R)$, for $0<R<R_0$, then $m=M=R_0H_0$. In this case, the region of uncertainly disappears and we get that rebound will occur if and only if $k_\mathrm{e(P)}<k_\mathrm{e(L)}$ and $k_\mathrm{e(P)}<k_\mathrm{out}(1+H_0R_0)$.
- If *H* is nonlinear but with $m=R_0H_0$ (in which case the lower bounding curve for *H*(*R*) is the tangent to the curve at $R=R_0$), then the red region where there is rebound for $k_\mathrm{out}>k_\mathrm{e(P)}/(1+R_0H_0)$ in Fig. 4 is preserved and the region of uncertainty is below this line. Conversely, if $M=R_0H_0$ (so that the upper bounding curve for *H*(*R*) is the tangent at $R=R_0$), then the green region where there is no rebound for $k_\mathrm{out}\le k_\mathrm{e(P)}/(1+R_0H_0)$ in Fig. 4 is preserved and the region of uncertainty is above this line.
- We note that when $k_\mathrm{e(L)}<k_\mathrm{e(P)}$ (the left green region in Fig. 6), then there is no rebound in the direct feedback model *for any feedback function H(R)*. As discussed above, when $k_\mathrm{e(L)}>k_\mathrm{e(P)}$, then the form of the feedback function determines whether or not rebound occurs.

In summary, the region where rebound occurs increases slightly to include some more values with $k_\mathrm{out}<k_\mathrm{e(P)}<k_\mathrm{e(L)}$ when direct feedback is introduced into the basic TMDD model.

## Analysis of the full TMDD with feedback model

In the full TMMD with feedback model, we have an extra differential equation that governs the dynamics of the moderator *w* and so our equations are now (20)–(23) with initial condition (24). As mentioned previously, the parameter $\epsilon$ influences the time scale for the response dynamics. If $\epsilon$ is small, the feedback responds slowly and we will show that this always leads to rebound. On the other hand, if $\epsilon$ is very large, the feedback responds very fast and we will show that rebound will occur in the same region as found with the direct feedback approximation.

### Dynamics of the receptor with feedback, without ligand

Before considering the full model with feedback, we first consider the effect of feedback on the dynamics of the receptor *y* (or *R* in dimensional form) in the absence of the ligand or product. We substitute $x=z=0$ into (20)–(23) to give the two equations

49 $$\begin{aligned} \dot{y}= & {} k_3(w-y),\quad y(0)=y_0 \end{aligned}$$

50 $$\begin{aligned} \dot{w}= & {} \epsilon (h(y)-w),\quad w(0)=w_0 \end{aligned}$$

Again, it is easily shown, using (h2) and (h3), that these equations have the unique steady state $y=w=1$, i.e. the baseline values. Below we will prove the following rebound result.

#### Theorem 5.1

For any $\epsilon \in (0,\infty )$, there are always some initial conditions with $y(0)<1$ which give rise to trajectories with rebound.

First we consider the linear stability of the baseline state as this gives the local behaviour near to the baseline. The Jacobian matrix evaluated at this steady state is given by

51 $$\begin{aligned} J_0=\left[ \begin{array}{ll} -k_3 &{}\quad k_3\\ -\epsilon h_0 &{}\quad -\epsilon \end{array}\right] \end{aligned}$$

(recalling that $h_0=-h'(1)\ge 0$). The eigenvalues of $J_0$ can be found as solutions of the quadratic characteristic polynomial derived from this matrix. The discriminant of this quadratic equation is

$$\begin{aligned} D{:}{=}\epsilon ^2-2k_3(1+2h_0)\epsilon +k_3^2. \end{aligned}$$

and the eigenvalues are

52 $$\begin{aligned} \lambda _3= & {} \frac{1}{2}\left( -(k_3+\epsilon ) - \sqrt{D}\right) = \frac{1}{2}\left( -(k_3+\epsilon )- \sqrt{(k_3-\epsilon )^2-4\epsilon k_3h_0}\right) , \end{aligned}$$

53 $$\begin{aligned} \lambda _4= & {} \frac{1}{2}\left( -(k_3+\epsilon ) + \sqrt{D}\right) = \frac{1}{2}\left( -(k_3+\epsilon )+ \sqrt{(k_3-\epsilon )^2-4\epsilon k_3h_0}\right) , \end{aligned}$$

while the eigenvectors are of the form

54 $$\begin{aligned} v_i = (k_3,k_3+\lambda _i), \quad \hbox {for}\quad i=3,4. \end{aligned}$$

Note that in this section, the definition of the eigenvalue $\lambda _3$ is different from that used in the previous section. The discriminant *D* is itself a quadratic function of $\epsilon$ which has two positive roots given by

55 $$\begin{aligned} \epsilon _1^\pm =k_3\left( 1+2h_0\pm \sqrt{4h_0(1+h_0)}\right) . \end{aligned}$$

Clearly the discriminant is negative between these roots and positive elsewhere, and so we have the following result.

#### Lemma 5.2

The Eqs. (49)–(50) have a unique steady state at $y=w=1$ and the eigenvalues $\lambda _3$ and $\lambda _4$ of the Jacobian matrix $J_0$ are

- both real and negative if $0<\epsilon \le \epsilon _1^-$ or if $\epsilon \ge \epsilon _1^+$;
- a complex conjugate pair with negative real part if $\epsilon _1^-<\epsilon <\epsilon _1^+$.

Furthermore, when $\epsilon \rightarrow \infty$, $\lambda _3\rightarrow -\infty$ and $\lambda _4\rightarrow \lambda _\infty =-k_3(1+h_0)$.

**Fig. 7:**
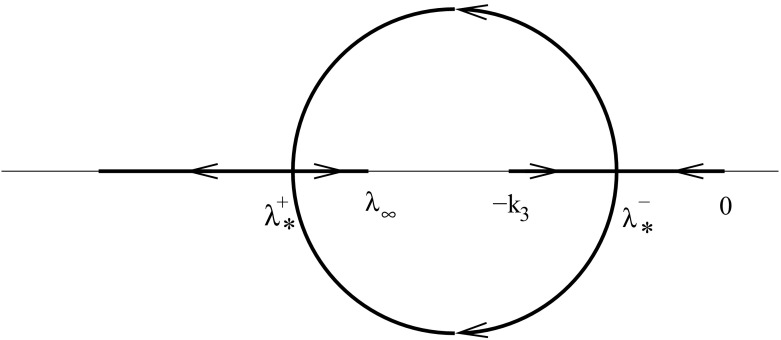
Movement of the eigenvalues $\lambda _3$ and $\lambda _4$ in the complex plane for increasing $\epsilon$ (indicated by the direction of the arrows)

For a proof of the last statement, see Appendix A. Lemma 5.2 is summarised in Fig. 7 with the direction of movement of the eigenvalues indicated corresponding to increasing $\epsilon$.

When $\epsilon =0$, we see that $\lambda _3=-k_3$ and $\lambda _4=0$. As $\epsilon$ increases from zero, the two eigenvalues move towards each other and collide on the negative real axis when $\epsilon =\epsilon _1^-$. After this collision, the eigenvalues become complex with $\mathrm{Re}(\lambda _{3,4})=-(k_3+\epsilon )/2$, which decreases monotonically with $\epsilon$. As $\epsilon$ increases further, the two complex eigenvalues collide on the real axis when $\epsilon =\epsilon _1^+$ and become real again. As $\epsilon$ increases from $\epsilon _1^+$ to $\infty$, $\lambda _4$ increases from the point of collision to $-k_3(1+h_0)$ and $\lambda _3$ decreases to $-\infty$ from this point. At the collision values $\epsilon =\epsilon _1^\pm$, the eigenvalues $\lambda _3=\lambda _4$ are given by

56 $$\begin{aligned} \lambda _*^\pm =-\frac{1}{2}(k_3+\epsilon _1^\pm ) =-k_3\left( 1+h_0\pm \sqrt{h_0(1+h_0)}\right) . \end{aligned}$$

Note that this implies that $\lambda _*^+<\lambda _\infty <\lambda _*^-$, as $\lambda _\infty =-k_3(1+h_0)$.

Lemma 5.2 implies linear stability, but a stronger stability result can be proved. The proof of this result uses a Lyapunov function and is given in Appendix A.

#### Lemma 5.3

In the region of ${\mathbb {R}}^2$ defined by $y,w\ge 0$, the steady state $y=w=1$ of Eqs. (49), (50) is globally asymptotically stable when $\epsilon >0$.

Thus the trajectory for all non-negative initial conditions will eventually approach the steady state $(y,w)=(1,1)$ and Theorem 5.1 can be proved by analysing the solutions close to the steady states. The following lemma gives the detailed results, with the proof again in Appendix A.

#### Lemma 5.4

- If $0<\epsilon {{{}\le \epsilon _1^-}}$ and $y(0)<1$, then rebound in Eqs. (49), (50) will occur for all $w(0) {{{}>1}}$ and for some values of $w(0) {{{}\le 1}}$.
- If $\epsilon _1^-<\epsilon _1<\epsilon _1^+$ then rebound occurs for any initial condition with $y(0)<1$.
- If $\epsilon _1^+ {{{}\le \epsilon <\infty }}$ and $y(0)<1$, then rebound in Eqs. (49), (50) will occur for some initial conditions which satisfy $w(0)>1$.

Having established the effect of the feedback on the behaviour of the receptor without ligand and product, we next consider the effect of adding the ligand.

### Invariance, steady states and stability

Before considering rebound, we first state invariance and stability results for the full TMDD system with feedback, similar to the results in Sect. 4.1 for the TMDD system with direct feedback. The proofs of the statements in this section can be found in Appendix A.

The first three of our variables are related to physical quantities and so must be non-negative and we have also assumed that the feedback variable *w* is non-negative. Therefore, we now establish the invariance of the region with $x,y,z,w\ge 0$ for Eqs. (20)–(23).

#### Lemma 5.5

The region of ${\mathbb {R}}^4$ defined by $x,y,z,w\ge 0$ is invariant under the flow of Eqs. (20)–(23) when $\epsilon >0$.

As with our previous model, this again shows that none of the variables can go negative, which is an essential property of a good model. We now consider the steady states of Eqs. (20)–(23).

#### Lemma 5.6

In the region of ${\mathbb {R}}^4$ defined by $x,y,z,w\ge 0$, there is a unique steady state of Eqs. (20)–(23), given by

57 $$\begin{aligned} x=0,\quad z=0,\quad y=1,\quad w=1. \end{aligned}$$

The Jacobian matrix obtained from Eqs. (20)–(23) evaluated at the steady state solution (57) is given by

58 $$\begin{aligned} \left( \begin{array}{cccc} -(k_1+1) &{}\quad \mu k_2 &{}\quad 0 &{}\quad 0\\ \frac{1}{\mu } &{}\quad -(k_2+k_4) &{}\quad 0 &{}\quad 0\\ -\frac{1}{\mu } &{}\quad k_2 &{}\quad -k_3 &{}\quad k_3\\ 0 &{}\quad 0 &{}\quad -\epsilon h_0 &{}\quad -\epsilon \end{array}\right) \end{aligned}$$

(recalling that $h_0=-h'(1)$). The eigenvalues of this matrix can be found from the top left $2\times 2$ matrix and the bottom right $2\times 2$ matrix. From the top left matrix, we obtain the eigenvalues $\lambda _1$ and $\lambda _2$ that we had previously, given by (27) and (28). The bottom right matrix is the same as $J_0$, defined in (51), and so the eigenvalues of this matrix are $\lambda _3$ and $\lambda _4$ as defined in (52) and (53). The corresponding eigenvectors are given by

59 $$\begin{aligned} v_i=\left( \begin{array}{c}\mu (k_2+k_4+\lambda _i) \\ 1 \\ \displaystyle -\frac{(k_4+\lambda _i)(\epsilon +\lambda _i)}{(k_3+\lambda _i)(\epsilon +\lambda _i)+\epsilon k_3h_0}\\ \displaystyle \frac{\epsilon h_0(k_4+\lambda _i)}{(k_3+\lambda _i)(\epsilon +\lambda _i)+\epsilon k_3h_0} \end{array}\right) ,\quad i=1,2;\quad v_i=\left( \begin{array}{c}0\\ 0\\ k_3\\ k_3+\lambda _i\end{array}\right) ,\quad i=3,4. \end{aligned}$$

We now immediately get the linear stability of the steady state solution as all eigenvalues $\lambda _i$ are strictly negative.

#### Lemma 5.7

The steady state (57) of Eqs. (20)–(23) is linearly stable when $\epsilon >0$.

This result implies that all trajectories with initial conditions *sufficiently close* to the steady state will converge to it. On its own , it does not guarantee convergence for all initial conditions. However, for this model, a stronger stability result can again be proved.

#### Lemma 5.8

The steady state $(x,z,y,w)=(0,0,1,1)$ of Eqs. (20)–(23) is globally asymptotically stable when $\epsilon >0$.

This result ensures that for any non-negative initial conditions and for all positive parameter values, each of the variables will converge to their unique steady state value, as with the previous model.

The proofs of these two results are again in Appendix A.

### Rebound

In this section, we focus on the analysis of rebound in the latter stages of the evolution, i.e., the approach to the steady state. The linearised system will play an important role in this, but first we consider the limiting case $\epsilon =0$ and derive some estimates on the initial behaviour of the moderator.

For $\epsilon =0$, the full TMDD equations with feedback via a moderator, i.e., (20)–(23), reduce to

$$\begin{aligned} \dot{x}= & {} -k_1 x - xy + \mu k_2 z \\ \dot{z}= & {} \frac{xy}{\mu }- \left( k_2 + k_4\right) z \\ \dot{y}= & {} k_3 (w - y) - \frac{xy}{\mu } + k_2 z\\ \dot{w}= & {} 0 \end{aligned}$$

Hence *w* is constant. With the initial condition (24), this implies $w=1$ and the system reduces to the TMDD equation without feedback as studied in our previous paper (Aston et al. 2014). However, if *w* has a different initial condition, say $w=w_0\ne 1$, then the system will converge to the steady state $(x,z,y,w)=(0,0,w_0,w_0)$. Within the hyperplane $w=w_0$, this equilibrium is an attractor. Thus if $w_0>1$, then the receptor will converge to a value above the original baseline. Note that in this case, we have $\lambda _4=0$, corresponding to the invariance of the moderator *w* and the line of steady states $(0,0,w_0,w_0)$.

For $\epsilon >0$ and the initial condition (24), we can show that initially the moderator *w* will increase to values above its baseline and hence the production of the receptor will be stimulated.

#### Lemma 5.9

For $\epsilon >0$ and $h_0>0$, there exists a $\tau ^*>0$ such that $w(\tau )>1$ for all $\tau \in (0,\tau ^*)$.

#### Proof

The initial condition is $w(0)=1$ and since this is also the steady state value of *w*, we have $\dot{w}(0)=0$. Differentiating (23) and evaluating at $\tau =0$ gives

$$\begin{aligned} \ddot{w}(0)=\frac{\epsilon h_0}{\mu }>0 \end{aligned}$$

and the result follows immediately from this. $\square$

The initial conditions give that $\dot{y} (0)= -\frac{1}{\mu }$, hence initially *y* will decrease and take values smaller than its baseline value 1. The following lemma implies that if there is no rebound, then $w(\tau )$ must be larger than 1 for all time. This observation will be used several times later to derive a contradiction on the assumption that there is no rebound.

#### Lemma 5.10

If $\epsilon >0$, $h_0>0$ and $0\le y(\tau )\le 1$ for all $\tau \ge 0$, then $w(\tau )>1$ for $\tau >0$.

#### Proof

Assume that $\epsilon >0$, $h_0>0$ and $0\le y(\tau )\le 1$ for all $\tau \ge 0$. We evaluate $\dot{w}$ along the hyperplane $w=1$ giving

$$\begin{aligned} \dot{w}|_{w=1}=\epsilon (h(y)-1). \end{aligned}$$

By hypothesis (h3), $h(y)>1$ if $y<1$ and so $\dot{w}|_{w=1}>0$ in this case. Since $w(\tau )>1$ for sufficiently small $\tau$ by Lemma 5.9, this result shows that $w(\tau )$ cannot touch or cross the line $w=1$ when $y(\tau )<1$.

Next we will show by contradiction that $y(\tau )=1=w(\tau )$ is impossible too. We assume that there is some $\tilde{\tau }>0$ such that $w(\tilde{\tau })=1$ with $w(\tau )>1$ for $\tau \in (0,\tilde{\tau })$, i.e. $\tilde{\tau }$ is the first moment that $w(\tau )$ equals one, and that $y(\tilde{\tau })=1$ also. Using the model equations (20)–(23), we obtain

$$\begin{aligned} \dot{y}(\tilde{\tau })= & {} -\left( \frac{x(\tilde{\tau })}{\mu } -k_2 z(\tilde{\tau })\right) , \quad \dot{w}(\tilde{\tau }) =0, \quad \ddot{w}(\tilde{\tau }) = -\epsilon h_0 \dot{y}(\tilde{\tau }),\\ \dddot{w}(\tilde{\tau })= & {} \epsilon [h''(1)(\dot{y}(\tilde{\tau }))^2-h_0\ddot{y}(\tilde{\tau })-\ddot{w}(\tilde{\tau })]. \end{aligned}$$

Thus, using Taylor series, *y* and *w* near to $\tilde{\tau }$ are given by

$$\begin{aligned} y(\tilde{\tau }+\hat{\tau })= & {} 1+\dot{y}(\tilde{\tau })\,\hat{\tau } + \frac{\ddot{y}(\tilde{\tau })}{2}\,\hat{\tau }^2 +\mathcal {O}(\hat{\tau }^3),\\ w(\tilde{\tau }+\hat{\tau })= & {} 1-\frac{\epsilon h_0}{2}\,\ddot{y}(\tilde{\tau })\,\hat{\tau }^2-\frac{\epsilon h_0}{6}\,\ddot{y}(\tilde{\tau })\,\hat{\tau }^3+\mathcal {O}(\hat{\tau }^4). \end{aligned}$$

The assumption $w(\tau )>1$ for $\tau <\tilde{\tau }$ and $y(\tau )\le 1$ for all $\tau$ together with $h_0>0$ imply that $\dot{y}(\tilde{\tau })=0$. Looking at the expressions for the derivatives at $\tilde{\tau }$, this gives $x(\tilde{\tau })=\mu k_2z(\tilde{\tau })$, $\ddot{w}(\tilde{\tau })=0$ and $\dddot{w}(\tilde{\tau })=-\epsilon h_0 \ddot{y}(\tilde{\tau })$. Going to the next order in the Taylor series, since $y(\tau )\le 1$, then we must have that $\ddot{y}(\tilde{\tau })\le 0$. If $\ddot{y}(\tilde{\tau })<0$ then this implies that $w(\tilde{\tau }+\hat{\tau })<1$ for $\hat{\tau }<0$ and $|\hat{\tau }|$ sufficiently small, which contradicts our earlier assumption on *w*. Thus, the only possibility is that $\ddot{y}(\tilde{\tau })=0$. Using the relation $x(\tilde{\tau })= \mu k_2 z(\tilde{\tau })$ we then find that

$$\begin{aligned} \ddot{y}(\tilde{\tau }) = k_2(k_1-k_4)z(\tilde{\tau }) \end{aligned}$$

and so this second derivative is zero if either $z(\tilde{\tau })=0$ or $k_1=k_4$. If $z(\tilde{\tau })=0$, then $x(\tilde{\tau })=\mu k_2 z(\tilde{\tau })=0$ too. As we also have $y(\tilde{\tau })=1=w(\tilde{\tau })$, this would imply that the solution is at the equilibrium at $t=\tilde{\tau }$. Uniqueness of solutions gives that this is not possible. With the alternative option $k_1=k_4$, evaluating $\dot{x}-\mu k_2 \dot{z}$, $\dot{y}$ and $\dot{w}$ on the line

$$\begin{aligned} \{x=\mu k_2 z, \, y=1, \,w=1\} \end{aligned}$$

shows that this line is invariant under the dynamics. At $t=0$, $x(0)-\mu k_2 z(0)=1$, hence initially the solution is not on this line and therefore it can never be on the line in finite time, which rules out the possibility that $k_1=k_4$ and we have come to a contradiction. We therefore conclude that $w(\tilde{\tau })\ne 1$ and so it follows that $w(\tau )>1$ for all $\tau >0$. $\square$

#### Corollary 5.11

If $\epsilon >0$, $h_0>0$ and $w(t^*)\le 1$ for some $t^*>0$, then rebound occurs.

#### Proof

This result is simply the converse of Lemma 5.10. $\square$

The observation that initially the moderator will be larger than 1, combined with the fact that for $\epsilon =0$, the initial condition $w(0)>1$ leads to rebound, makes it likely that rebound will happen for all small values of $\epsilon$. This is indeed the case as will be shown below.

#### Relative position of the eigenvalues

To analyse rebound in the latter stages of the evolution, we focus on solutions near to the baseline state. We are especially interested in the eigenvalue of the Jacobian matrix (58) with real part closest to zero, since generically trajectories approach the steady state tangent to the eigenvector corresponding to this eigenvalue. We note that the eigenvalues $\lambda _1$ and $\lambda _2$ are always real, depend on $k_1$, $k_2$ and $k_4$, do not depend on $\epsilon$ and $k_3$, and that $\lambda _2<\lambda _1$. Furthermore, for $k_2$ and $k_4$ fixed, the eigenvalue $\lambda _1(k_1,k_2,k_4)$ is monotonic decreasing in $k_1$ and

60 $$\begin{aligned} \lambda _1(0,k_2,k_4)= & {} {\textstyle -\frac{1+k_2+k_4 - \sqrt{(1+k_2+k_4)^2-4k_4}}{2}}; \quad \lambda _1(k_4,k_2,k_4) = -k_4;\nonumber \\&\lim _{k_1\rightarrow \infty }\lambda _1(k_1,k_2,k_4) =-(k_2+k_4). \end{aligned}$$

On the other hand, the eigenvalues $\lambda _3$ and $\lambda _4$ depend on $k_3$ and $\epsilon$, do not depend on $k_1$, $k_2$ and $k_4$, and $\mathrm {Re}(\lambda _3)\le \mathrm {Re}(\lambda _4)$. Thus, the eigenvalue with real part closest to zero will be either $\lambda _1$ or $\lambda _4$. As observed before, $\lambda _1$ does not depend on $\epsilon$, and for small $\epsilon$ we have

$$\begin{aligned} \lambda _4=-(1+h_0)\epsilon +O(\epsilon ^2). \end{aligned}$$

**Fig. 8:**
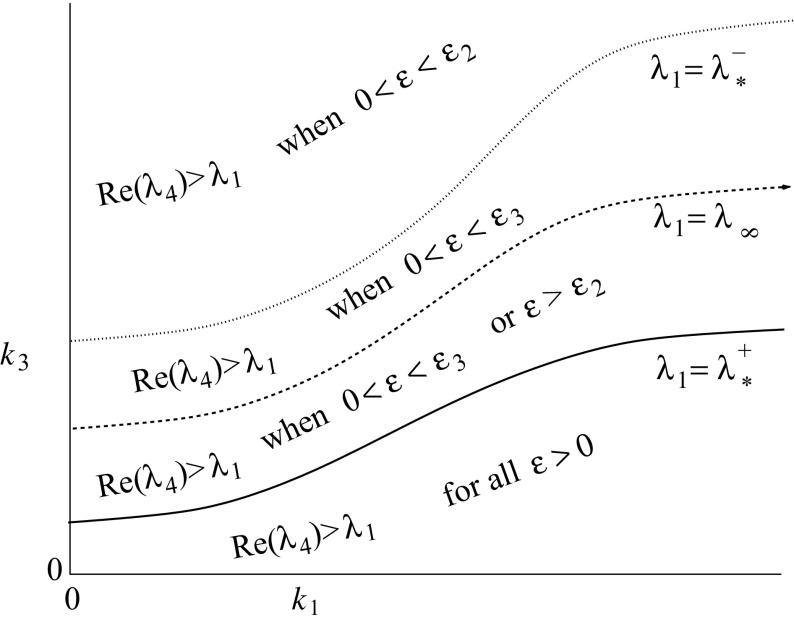
The relative positioning of the eigenvalues in the $k_1$–$k_3$ plane with $k_2$ and $k_4$ fixed. Recall that $\lambda _\infty (k_3) = -k_3(1+h_0)$ and $\lambda _*^\pm (k_3) =-k_3(1+h_0\pm \sqrt{h_0(1+h_0)})$, thus the three curves are relations of the form $k_3=\lambda _1(k_1,k_2,k_4)/ f(h_0)$, with $f(h_0)$ the appropriate function

Thus, for sufficiently small $\epsilon$, the eigenvalue closest to zero will be $\lambda _4$. To describe how this changes when $\epsilon$ increases, we consider four $\lambda _1$ intervals that are defined in terms of $\lambda _*^\pm$ (see (56)) and $\lambda _\infty$ (see Lemma 5.2)—a summary is sketched in Fig. 8:

- $$\lambda ^-_*(k_3)\le \lambda _1(k_1,k_2,k_4)<0$$ (i.e., $$k_3\ge \frac{-\lambda _1}{1+h_0- \sqrt{h_0(1+h_0)}}$$):
  Now $\lambda _1$ is in the interval where $\lambda _4$ takes real values, hence the eigenvalues $\lambda _4$ and $\lambda _1$ cross at the point where $\lambda _4=\lambda _1$ and solving this equation for $\epsilon$ gives
  61 $$\begin{aligned} \epsilon= & {} \epsilon _2(\lambda _1,k_3):=-\lambda _1 \left( \frac{\lambda _1+k_3}{\lambda _1+k_3(1+h_0)}\right) = \epsilon _1^-(k_3) - \frac{(\lambda _1-\lambda _*^-)^2}{\lambda _1-\lambda _\infty }\nonumber \\= & {} \epsilon _1^+(k_3) +\frac{ (\lambda _1-\lambda _*^+)^2}{\lambda _\infty -\lambda _1} . \end{aligned}$$
  Note that $\lambda _1+k_3(1+h_0)=\lambda _1-\lambda _\infty \ne 0$ in this interval and so $\epsilon _2(\lambda _1,k_3)$ is well defined. Thus $\epsilon _2(\lambda _1,k_3)= \epsilon _1^-(k_3)$, when $\lambda _1=\lambda _*^-$. Altogether, we conclude that in this $\lambda _1$ interval, we have that $\lambda _4>\lambda _1$ if $0<\epsilon <\epsilon _2(\lambda _1,k_3).$
- $$\lambda _\infty (k_3)\le \lambda _1(k_1,k_2,k_4)\le \lambda ^-_*(k_3)$$ (i.e., $$\frac{-\lambda _1}{1+h_0}\le k_3\le \frac{-\lambda _1}{1+h_0-\sqrt{h_0(1+h_0)}}$$):
  Here $\lambda _1$ is in the interval where $\lambda _4$ takes complex values, hence for $\epsilon <\epsilon _1^-(k_3)$, $\lambda _4$ is real and will be the eigenvalue closest to zero. At $\epsilon =\epsilon _1^-(k_3)$, $\lambda _4$ and $\lambda _3$ merge and become a complex conjugate pair. For $\epsilon$ just larger than $\epsilon _1^-$, the real part of the complex pair will be closest to zero and this will be the case until the real part of the complex eigenvalues crosses $\lambda _1$. This occurs when $\mathrm{Re}(\lambda _{3,4})=\lambda _1$ and since $\mathrm{Re}(\lambda _{3,4})= -(k_3+\epsilon )/2$, this crossing occurs at $\epsilon =\epsilon _3$, where
  $$\begin{aligned} \epsilon _3(\lambda _1,k_3):=-(k_3+2\lambda _1) \end{aligned}$$
  Since $\epsilon ^\pm _1 = -(k_3+2\lambda ^\pm _*)$, it follows immediately that
  62 $$\begin{aligned} \epsilon _1^-(k_3)<\epsilon _3(\lambda _1,k_3)<\epsilon _1^+(k_3), \hbox { for } \lambda _*^+<\lambda _1<\lambda ^-_*. \end{aligned}$$
  Note that $\epsilon _3(\lambda _1,k_3)= \epsilon _1^-(k_3)=\epsilon _2(\lambda _1,k_3)$, when $\lambda _1=\lambda _*^-$. Thus in this interval, we have that $\mathrm {Re}(\lambda _4)>\lambda _1$ for $0<\epsilon <\epsilon _3(\lambda _1,k_3)$.
- $$\lambda ^+_*(k_3)\le \lambda _1(k_1,k_2,k_4)<\lambda _\infty (k_3)$$ (i.e., $$\frac{-\lambda _1}{1+h_0+ \sqrt{h_0(1+h_0)}}\le k_3< \frac{-\lambda _1}{1+h_0}$$):
  Now $\lambda _1$ is in the interval where $\lambda _4$ takes both real and complex values. If $\epsilon >\epsilon _1^+(k_3)$, then $\lambda _4$ is real and we can use the calculation in the first point to conclude that $\lambda _4>\lambda _1$ if $\epsilon >\epsilon _2(\lambda _1,k_3)$. If $\epsilon _1^-(k_3)<\epsilon <\epsilon _1^+(k_3)$, then $\lambda _4$ is complex and the calculation above gives that $\mathrm {Re}(\lambda _4)>\lambda _1$ if $0<\epsilon <\epsilon _3(\lambda _1,k_3)$. Note that $\epsilon _2(\lambda _1,k_3)>\epsilon _1^+(k_3)>\epsilon _3(\lambda _1,k_3)$ in the open $\lambda _1$ interval and that $\epsilon _3(\lambda _1,k_3)$ and $\epsilon _2(\lambda _1,k_3)$ collide with $\epsilon _1^+(k_3)$ when $\lambda _1=\lambda ^+_*(k_3)$. Furthermore, $\epsilon _2(\lambda _1,k_3)\rightarrow \infty$ for $\lambda _1\rightarrow \lambda _\infty$. So altogether we have that in this interval
  $$\begin{aligned} \mathrm {Re}(\lambda _4)>\lambda _1 ~\hbox { if }~ 0<\epsilon <\epsilon _3(\lambda _1,k_3) ~\hbox { or }~ \epsilon >\epsilon _2(\lambda _1,k_3). \end{aligned}$$
- $$\lambda _1(k_1,k_2,k_4)<\lambda ^+_*(k_3)$$ (i.e., $$k_3< \frac{-\lambda _1}{1+h_0+ \sqrt{h_0(1+h_0)}}$$):
  Since $\mathrm {Re}(\lambda _4)\ge \lambda ^+_*(k_3)$, we see immediately that in this interval $\mathrm {Re}(\lambda _4)> \lambda _1$ for all $\epsilon >0$.

To visualise this information, we have sketched the $\lambda _1$ intervals in the $k_1$-$k_3$ plane in Fig. 8.

#### Rebound in the latter stages of the evolution

Now that we have determined the positioning of the eigenvalues, we can state when rebound occurs in the latter stages of the evolution.

##### Theorem 5.12

Define *m* as in Theorem 4.10, i.e., $m=\inf \{\frac{h(y)-1}{y-1}\mid 0\le y <1 \}$ and define $f_*^+(h_0) = 1+h_0+\sqrt{h_0(1+h_0)}$, hence $\lambda _*^+=-k_3f_*^+(h_0)$.

(i) If $k_3<\min \left( \frac{-\lambda _1}{f_*^+(h_0)}, \frac{k_4}{1+m}\right)$, then rebound occurs generically for $0<\epsilon <\epsilon _1^+{{(k_3)}}$.
(ii) If $k_1>k_4$ and
  - $\frac{-\lambda _1}{f^+_*(h_0)}< k_3 <\min \left( \frac{-\lambda _1}{1+h_0},\frac{k_4}{1+m}\right)$, then rebound occurs generically for $0<\epsilon <\epsilon _2{{(\lambda _1,k_3)}}$;
  - $k_3> \min \left( \frac{-\lambda _1}{1+h_0},\frac{k_4}{1+m}\right)$, then rebound occurs generically for all $\epsilon >0$.
(iii) If $k_1<k_4$ and
  - $-\frac{\lambda _1}{f_*^+(h_0)}<k_3\le -\lambda _1$ then rebound occurs generically for $0<\epsilon <\epsilon _3{{(\lambda _1,k_3)}}$;
  - $k_3>-\lambda _1$ then rebound occurs generically for $0<\epsilon <-\lambda _1$.

**Fig. 9:**
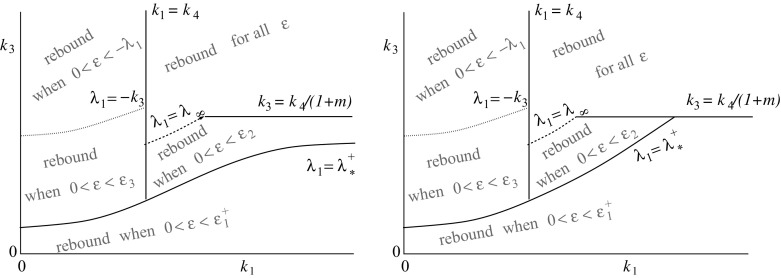
Rebound overview, on the left for $k_2\le k_4\,\frac{f_*^+-(1+m)}{1+m}$ and on the right for $k_2>k_4\,\frac{f_*^+-(1+m)}{1+m}$. Note that $f^+_*(h_0)>1+h_0\ge 1+m$. Recall that $\lambda _*^+=-k_3f_*^+(h_0)$ and $\lambda _\infty =-k_3(1+h_0)$

These results are summarised in Fig. 9.

The proof of this theorem will be given via two lemmas which determine when the eigenvalue closest to zero can be associated with rebound. First we consider the case that the eigenvalue $\lambda _4$ (or its real part if complex) is closest to zero.

##### Lemma 5.13

If the eigenvalue $\lambda _4$ (or its real part if complex) is closest to zero and

- $$0<\epsilon <\epsilon _1^+$$ or
- $$\epsilon \ge \epsilon _1^+$$, and $$k_3>\frac{k_4}{1+m}$$,

then rebound occurs (generically only for $\epsilon _1^-<\epsilon <\epsilon _1^+$).

The proof of this lemma can be found in Appendix B.

**Table 1:** Summary of results obtained by applying Lemmas 5.13, 5.14 to the eigenvalue categories described in Sect. 5.3.1. The ticks indicate that rebound occurs in the given intervals

Region	$\mathrm{Re}(\lambda _4)>\lambda _1$	Conditions for rebound	$\mathrm{Re}(\lambda _4)<\lambda _1$	Conditions for rebound
$\lambda _*^-\le \lambda _1<0$	$0<\epsilon<\epsilon _2(\, <\epsilon _1^+)$	$\checkmark$	$\epsilon >\epsilon _2$	$k_1>k_4$ or $\epsilon _2<\epsilon <-\lambda _1$
$\lambda _\infty \le \lambda _1\le \lambda _*^-$	$0<\epsilon<\epsilon _3(\,<\epsilon _1^+)$	$\checkmark$	$\epsilon >\epsilon _3$	$k_1>k_4$ or $\epsilon _3<\epsilon <-\lambda _1$ if $\lambda _1>-k_3$
$\lambda _*^+\le \lambda _1<\lambda _\infty$	$0<\epsilon <\epsilon _3(\,\le \epsilon _1^+)$ $\epsilon >\epsilon _2(\,\ge \epsilon _1^+)$	$\checkmark$ $k_3>\frac{k_4}{1+m}$	$\epsilon _3<\epsilon <\epsilon _2$	$k_1>k_4$
$\lambda _1<\lambda _*^+$	$\epsilon >0$	$0<\epsilon <\epsilon _1^+$ or $\epsilon \ge \epsilon _1^+$ if $k_3>\frac{k_4}{1+m}$	−	−

Next we consider the case that the eigenvalue $\lambda _1$ is closest to zero.

##### Lemma 5.14

If $\lambda _1$ is the eigenvalue closest to zero, then rebound occurs generically if $k_1>k_4$ or if $\epsilon <-\lambda _1$.

Again, the proof of this lemma can be found in Appendix B.

With the lemmas above, we are ready to prove Theorem 5.12.

##### Proof of Theorem 5.12

The results in this Theorem are essentially obtained by applying Lemmas 5.13 and 5.14 in each of the four regions described in Sect. 5.3.1. The results from this process are summarised in Table 1. The observations about the relative position of $\epsilon _2$, $\epsilon _3$ and $\epsilon ^+$ in the various regions follow from (61) and (62). In the regions $\lambda _*^+<\lambda _1<\lambda _*^-$, the case $\mathrm{Re}(\lambda _4)<\lambda _1$ leads to the range $\epsilon _3<\epsilon <-\lambda _1$. This range is not empty only when $\epsilon _3<-\lambda _1$. The definition of $\epsilon _3$ shows that this condition holds if $\lambda _1>-k_3$, which is why this condition is included in the second row. For the third row, we observe that $\lambda _\infty =-k_3(1+h_0)<-k_3$, so the range $\epsilon _3<\epsilon <-\lambda _1$ is always empty if $\lambda _1<\lambda _\infty$.

The two columns containing conditions for rebound in Table 1 can be combined in some cases, as shown in Table 2, where we separate out two regions in the parameter plane defined by $k_1\le k_4$ and $k_1>k_4$. For the third row, we have used that $\lambda _1(k_1,k_2,k_4)$ is monotonic decreasing in $\lambda _1$. Hence if $k_1\le k_4$, then $\lambda _1(k_1,k_2,k_4)\ge \lambda _1(k_4,k_2,k_4)=-k_4$ (see (60)). Thus if $\lambda _1<\lambda _\infty =-k_3(1+h_0)$ and $k_1\le k_4$, then $k_3<-\frac{\lambda _1}{1+h_0}\le \frac{k_4}{1+h_0}\le \frac{k_4}{1+m}$.

**Table 2:** Summary of conditions for rebound derived from Table 1

Region	$k_1\le k_4$	$k_1>k_4$
$\lambda _*^-\le \lambda _1<0$	$0<\epsilon <\epsilon _2$, $\epsilon _2<\epsilon <-\lambda _1$	$0<\epsilon <\epsilon _2$, $\epsilon _2<\epsilon$
$\lambda _\infty \le \lambda _1\le \lambda _*^-$	$0<\epsilon <\epsilon _3$, $\epsilon _3<\epsilon <-\lambda _1$ if $\lambda _1>-k_3$	$0<\epsilon <\epsilon _3$, $\epsilon _3<\epsilon$
$\lambda _*^+\le \lambda _1<\lambda _\infty$	$0<\epsilon <\epsilon _3$	$0<\epsilon <\epsilon _3$, $\epsilon _3<\epsilon <\epsilon _2$, $\epsilon >\epsilon _2$ if $k_3>\frac{k_4}{1+m}$
$\lambda _1<\lambda _*^+$	$0<\epsilon <\epsilon _1^+$, $\epsilon \ge \epsilon _1^+$ if $k_3>\frac{k_4}{1+m}$	$0<\epsilon <\epsilon _1^+, \epsilon \ge \epsilon _1^+ \text { if } k_3> \frac{k_4}{1+m}$

We note in Table 2 that in many cases, there are neighbouring intervals with only the boundary point between them missing. Thus, filling in these specific points (which are proved below) gives the results shown in Table 3. The results in Theorem 5.12 are obtained by converting the eigenvalue ranges in Table 3 into conditions involving the parameters, using that $\lambda _\infty =-k_3(1+h_0)$ and $\lambda _*^+=-k_3f_*^+(h_0)$.

**Table 3:** Summary of final conditions for rebound

Region	$k_1\le k_4$	$k_1>k_4$
$\lambda _*^-\le \lambda _1<0$	$0<\epsilon <-\lambda _1$	$\epsilon >0$
$\lambda _\infty \le \lambda _1\le \lambda _*^-$	$0<\epsilon <\epsilon _3$ or $0<\epsilon <-\lambda _1$ if $\lambda _1>-k_3$	$\epsilon >0$
$\lambda _*^+\le \lambda _1<\lambda _\infty$	$0<\epsilon <\epsilon _3$	$0<\epsilon <\epsilon _2$ or $\epsilon >0$ if $k_3>\frac{k_4}{1+m}$
$\lambda _1<\lambda _*^+$	$0<\epsilon <\epsilon _1^+$ or $\epsilon >0$ if $k_3>\frac{k_4}{1+m}$	

We now consider a number of special cases that fill in the gaps that allow us to go from Tables 2 to 3.

- $$\lambda _*^-<\lambda _1<0$$, $$\epsilon =\epsilon _2$$.
  When $\epsilon =\epsilon _2$, then $\lambda _1=\lambda _4$ and so there are two repeated eigenvalues that are the least negative. With some calculations, it can be seen that the eigenvectors $v_1$ and $v_4$ collide (the denominator $(k_3+\lambda _1)(\epsilon _3+\lambda _1)+\epsilon _2k_3h_0$ in the third and fourth entry vanishes, so the eigenvector has to be rescaled first to see this), hence we are in the case of an eigenvector and a generalised eigenvector. The eigenvector is $v_4=(0,0,k_3,k_3+\lambda _4)$ and generically solutions will align with this eigenvector. In the proof of Lemma B.1, it is shown that $k_3+\lambda _4>0$ when $\epsilon \le \epsilon _1^-$. Now $\epsilon _2<\epsilon _1^-$ for $\lambda _1>\lambda _*^-$ by (61) and this implies that the third and fourth components of $v_4$ have the same sign. The proof in Lemma B.1 now applies to prove that generically rebound occurs in this case.
- $$\lambda _1=\lambda _*^-$$, $$\epsilon =\epsilon _2=\epsilon _3=\epsilon _1^-$$.
  In this case there is triple eigenvector with one eigenvector $v_1=v_4=v_3 = (0,0,k_3,k_3+\lambda _4)$ together with two generalised eigenvectors. Again, generically solutions align with this eigenvector and, as above, the third and fourth entries have the same sign, thus and so rebound can be proved via a contradiction argument.
- $$\lambda _*^+<\lambda _1<\lambda _\infty $$, $$\epsilon =\epsilon _2$$ with $$k_3>\frac{k_4}{1+m}$$.
  This is similar to the first case as the two eigenvalues $\lambda _1$ and $\lambda _4$ coincide when $\epsilon =\epsilon _2$ with an eigenvector and a generalised eigenvector in this case. Generically solutions will align asymptotically with the eigenvector. The proof of Lemma B.2 can now be applied in this case.
- $$\lambda _*^+<\lambda _1<\lambda _*^-$$, $$\epsilon =\epsilon _3$$ with $$k_1>k_4$$ or $$k_3>-\lambda _1$$.
  In this case, the real part of the complex pair $\lambda _{3,4}$ equals the eigenvalue $\lambda _1$. Approaching the steady state via the complex pair leads to rebound. The proof of Lemma 5.14 also applies with $\epsilon =\epsilon _3$ provided that $\epsilon _3+\lambda _1<0$ and this occurs if $k_3>-\lambda _1$ and so rebound occurs if the steady state is approached tangent to $v_1$. If the approach occurs via a linear combination of the three eigenvectors, then clearly rebound will then occur in this case also. Hence we can conclude that rebound will occur generically in this case.

The rebound results that are summarised in Table 3 are shown in Fig. 9. There are two cases shown here, depending on whether or not the curve $\lambda _1=\lambda _*^+$ intersects the line $k_3=\frac{k_4}{1+m}$. Since $\lim _{k_1\rightarrow \infty }=-(k_2+k_4)$ (see (60)), if $k_2<k_4\frac{f_*^+-(1+m)}{1+m}$ then the curve $\lambda _1=\lambda _*^+$ is always below $k_3=\frac{k_4}{1+m}$. Also, as $\lambda _1(k_4,k_2,k_4)=-k_4$ (see (60)), then it can be shown that the line $k_3=\frac{k_4}{1+m}$ is above the line $\lambda _*^+$ at this point, and so the line can never go below the curve as the curve is monotonically increasing with $k_1$. $\square$

##### Remarks

- Some of the rebound results that we proved in Lemmas 5.13 and 5.14 hold only generically, and some cases always give rebound. However, for the sake of simplicity, we have not distinguished between these in Theorem 5.12 but simply refer to the existence of generic rebound.
- In Theorem 5.12, we have proved the existence of rebound in a variety of cases. However, it should be noted that we have not proved or disproved the existence of rebound in other parameter regions, and so we do not have results for all possible parameter values.
- From Theorem 5.12, we can conclude that there exists $\epsilon _0>0$ such that rebound occurs for all $\epsilon$ satisfying $0<\epsilon <\epsilon _0$ (see Fig. 9). Thus, for all parameter values, rebound occurs for sufficiently small $\epsilon >0$. This might seem to contradict the result for the limit $\epsilon =0$ (the no-feedback case) where there is a large region in the parameter plane for which there is no rebound. The explanation is that for those parameter values, the magnitude of the rebound decays to zero as $\epsilon$ goes to zero.

## Generalisations

Thus far, we have considered the basic TMDD model with feedback dynamics which is linear in the feedback moderator *F*. We now extend this model in two directions. First we extend the TMDD model to allow for more compartments and secondly we consider more general types of feedback dynamics. Below we show that some of the rebound results can be extended to these more general models.

### Generalised TMDD model with feedback via a moderator

In this section we consider a generalisation of the basic TMDD model to more compartments. It is assumed that the receptor is still only in one compartment. In the basic TMDD model, the variable *y* represents the normalised receptor in this compartment. Now we will assign *y* to represent either the normalised free receptor (as before) or it could be the normalised total amount of receptor. The normalised ligand-product components (*x* and *z*) of the basic TMDD model are generalised to a vector $\mathbf {x}$ that represents the ligand and product in the compartments. The generalised TMDD model with feedback becomes

63 $$\begin{aligned} \dot{\mathbf {x}}= & {} \mathbf {f}_1(\mathbf {x},y) \end{aligned}$$

64 $$\begin{aligned} \dot{y}= & {} k_3(w-y)+f_2(\mathbf {x},y) \end{aligned}$$

65 $$\begin{aligned} \dot{w}= & {} \epsilon (h(y)-w) \end{aligned}$$

where $\mathbf {x}\in \mathbb {R}^n$, $n\ge 1$ and $y,w\in \mathbb {R}$. We note that if $\mathbf{f}_1$ depends only on $\mathbf{x}$ but not on *y*, or if $f_2$ depends only on *y* but not on $\mathbf{x}$, then the coupling between $\mathbf{x}$ and *y* is in one direction only. In this case, the model could not be classed as a generalised TMDD model, although our results below still hold in these cases. We assume that the assumptions (h1)–(h4) on the feedback function *h*(*y*) hold and we make the following assumptions on the functions $\mathbf {f}_1$ and $f_2$:

- There exists an $\mathbf {x}_0\in \mathbb {R}^n$ such that the Eqs. (63)–(65) have the steady state solution $\mathbf {x}=\mathbf {x}_0$, $y=w=1$. This implies that $\mathbf {f}_1(\mathbf {x}_0,1)=\mathbf {0}$ and $f_2(\mathbf {x}_0,1)=0$. We also assume that this steady state is globally asymptotically stable.
- In the basic TMMD model, a change in the receptor level while the ligand and product are at baseline does not move the ligand or product away from baseline and we make a similar assumption for this model, i.e., $\mathbf {f}_1(\mathbf {x}_0,y)=0$ for all $y\ge 0$ which implies that $\frac{\partial \mathbf {f}_{1}}{\partial y}(\mathbf {x}_0,1)=0$. Similarly, the function $f_2$ is not affected by the receptor dynamics if the ligand and product are at baseline, i.e., $f_2(\mathbf {x}_0,y)=0$ for all $y\ge 0$ which implies that $\frac{\partial f_{2}}{\partial y}(\mathbf {x}_0,1)=0$. In fact, in both cases, all that we really require is the weaker derivative condition.
- The initial conditions are given by
  $$\begin{aligned} \mathbf {x}=\mathbf {x}_1,\quad y=w=1 \end{aligned}$$
  for some $\mathbf {x}_1\ne \mathbf {x}_0$ with $\mathbf {x}_1$ such that $f_2(\mathbf {x}_1,1)<0$. Hence initially the amount of receptor will decrease.
- The Jacobian matrix $D_{\mathbf {x}}\mathbf {f}_1(\mathbf {x}_0,1)$ has no zero or purely imaginary eigenvalues.

With these assumptions, the Jacobian matrix evaluated at the steady state is given by

$$\begin{aligned} J(\mathbf {x}_0,1,1)=\left[ \begin{array}{ccc} D_{\mathbf {x}}\mathbf {f}_{1}(\mathbf {x}_0,1) &{}\quad 0 &{}\quad 0\\ D_\mathbf {x}f_{2}(\mathbf {x}_0,1) &{}\quad -k_3 &{}\quad k_3\\ 0 &{} \quad -\epsilon h_0 &{}\quad -\epsilon \end{array}\right] \end{aligned}$$

Note that the condition $\frac{\partial \mathbf {f}_{1}}{\partial y}(\mathbf {x}_0,1)=0$ leads to a block lower triangular structure in this matrix. This implies that the eigenvalues of this matrix are the eigenvalues of the Jacobian matrix $D_{\mathbf {x}}\mathbf {f}_{1}(\mathbf {x}_0,1)$ and the eigenvalues of the $2\times 2$ bottom right matrix in $J(\mathbf {x}_0,1,1)$. The global asymptotic stability of the steady state $(\mathbf {x}_0,1,1)$ implies that all eigenvalues have negative or zero real part. Note that the eigenvalues of $D_{\mathbf {x}}\mathbf {f}_{1}(\mathbf {x}_0,1)$ do not depend on $\epsilon$ and that our final assumption implies that the eigenvalue closest to zero will have strictly negative real part. The $2\times 2$ bottom right matrix is the same as in the basic TMDD model with feedback. We denote its eigenvalues by $\lambda _n$ and $\lambda _{n-1}$, where the eigenvalue $\lambda _n$ corresponds to $\lambda _4$ earlier and $\lambda _{n-1}$ to $\lambda _3$. Thus $\lambda _n$ is the closest eigenvalue to zero for sufficiently small $\epsilon$.

We define

$$\begin{aligned} \tilde{\epsilon }_0=\min \{\epsilon _1^-,\tilde{\epsilon }_2\} \end{aligned}$$

where $\tilde{\epsilon }_2$ is the value of $\epsilon$ at which the eigenvalue $\lambda _n$ coincides with the eigenvalue closest to zero of the matrix $D_{\mathbf {x}}f_{1}(\mathbf {x}_0,1)$. Recall that $\epsilon _1^-$ is the value of $\epsilon$ for which $\lambda _n$ and $\lambda _{n-1}$ become a complex pair of eigenvalues. We then have the following result.

#### Theorem 6.1

If the above assumptions on $\mathbf {f}_1$ and $f_2$ hold, then rebound occurs generically for $0<\epsilon <\tilde{\epsilon }_0$.

#### Proof

The condition $f_2(\mathbf {x}_1,1)<0$ ensures that $\dot{y}(0)<0$, which is required in the proof of Lemma 5.9. The *w* equation is unchanged in this setting and so Lemma 5.10 also holds. The proof of Lemma B.1 also holds in this setting, although the exceptional case that the trajectory approaches the steady state tangent to a different eigenvector cannot be excluded outright in this more general case, and so we only have a generic result. $\square$

If the bottom right $2\times 2$ matrix in $J(\mathbf {x}_0,1,1)$ has complex eigenvalues with real part closest to zero of all the eigenvalues, then again there will be oscillation about the steady state in *y* as the steady state is approached and so rebound occurs infinitely often but with exponentially decreasing amplitude.

### Generalisation of the dynamics of the feedback

In our considerations so far, the feedback dynamics has been linear in the feedback moderator *F* (or *w* in the non-dimensional equations), see (14) or (23). In this section we will consider a more general form of feedback, and focus on the case of a small value of the parameter $\alpha$ ($\epsilon$). In particular, we again assume that the receptor equation is described by (13) and describe the dynamics of *F* by

66 $$\begin{aligned} \frac{dF}{dt}=\alpha G(R,F). \end{aligned}$$

Non-dimensionalising as before, we obtain a new equation for the feedback variable *w* ($=F$) given by

67 $$\begin{aligned} \dot{w}=\epsilon g(y,w), \end{aligned}$$

where

$$\begin{aligned} g(y,w)=G(R_0y,w) \end{aligned}$$

and $\epsilon$ is defined by (25). We make a number of assumptions regarding the function *g*:

1. $$g(1,1)=0$$
2. $$-g_1:=\frac{\partial g}{\partial y}(1,1)<0$$
3. $$-g_2:=\frac{\partial g}{\partial w}(1,1)<0$$

The assumption (h1’) ensures that the equations retain the steady state with $y=w=1$. Assumption (h2’) ensures that when the system is perturbed from the steady state by reducing *y*, which is what happens when the ligand is injected, then *w* will increase, resulting in greater production of the receptor. Finally, assumption (h3’) ensures that the steady state $w=1$ of (67), assuming that $y=1$ is held fixed, is stable.

We now show that some of the results obtained previously also hold in this more general setting. We again start by considering the Jacobian matrix of Eqs. (20)–(22), (67) evaluated at the steady state (57), which is given by

68 $$\begin{aligned} \left( \begin{array}{cccc} -(k_1+1) &{}\quad \mu k_2 &{}\quad 0 &{}\quad 0\\ \frac{1}{\mu } &{}\quad -(k_2+k_4) &{}\quad 0 &{}\quad 0\\ -\frac{1}{\mu } &{}\quad k_2 &{}\quad -k_3 &{}\quad k_3\\ 0 &{}\quad 0 &{}\quad -\epsilon g_1 &{}\quad -\epsilon g_2 \end{array}\right) \end{aligned}$$

#### Theorem 6.2

If assumptions $(h1')$, $(h2')$ and $(h3')$ hold, and if $g(y,1)>0$ for all $y\in [0,1)$, then there exists $\epsilon _0>0$ such that rebound occurs in the model given by Eqs. (20)–(22), (67) for all $\epsilon$ satisfying $0<\epsilon <\epsilon _0$.

#### Proof

We note that assumptions (h1$'$) and (h2$'$) imply that $g(y,1)>0$ for $y<1$ sufficiently close to 1. The extra assumption requires that this holds for all $y\in [0,1)$.

The proofs of Lemmas 5.9, 5.10 and B.1 can easily be adapted to this modified model. In particular, for Lemma 5.9, $\dot{w}(0)=0$ as before and in this case, we have

$$\begin{aligned} \ddot{w}(0)=-\frac{\epsilon }{\mu }\frac{\partial g}{\partial y}(1,1)=\frac{\epsilon }{\mu }g_1>0 \end{aligned}$$

and so Lemma 5.9 holds.

For Lemma 5.10, we note that

$$\begin{aligned} \dot{w}|_{w=1}=\epsilon g(y,1)>0 \end{aligned}$$

using our stated assumption. Combined with Lemma 5.9, this implies that $w(\tau )$ cannot touch or cross the line $w=1$ when $y(\tau )<1$. The second part of the proof of Lemma 5.10 shows by contradiction that $y(\tau )=w(\tau )=1$ is impossible and the same proof holds in this case with $h_0$ replaced by $g_1$.

The Jacobian matrix associated with Eqs. (49) and (67) (analogous to (51)) is given by

69 $$\begin{aligned} J_0=\left[ \begin{array}{ll}-k_3 &{}\quad k_3\\ -{\epsilon } g_1 &{}\quad -\epsilon g_2 \end{array}\right] \end{aligned}$$

which has eigenvalues $\lambda _3=-k_3$, $\lambda _4=0$ when $\epsilon =0$ as previously. These two eigenvalues collide when the discriminant of the characteristic polynomial is zero, and this first occurs when

$$\begin{aligned} \epsilon =\epsilon _1^-=\frac{k_3}{g_2^2}\left( 2g_1+g_2-\sqrt{4g_1(g_1+g_2)}\right) . \end{aligned}$$

With this definition of $\epsilon _1^-$, we claim that Lemma B.1 holds also. To see this, we note that the eigenvector $v_4$ given by (59) is the same in this case, but with $\lambda _4$ as the eigenvalue of (69) that is closest to zero. We now have

$$\begin{aligned} k_3+\lambda _4=\frac{1}{2}\left( k_3-\epsilon g_2+\sqrt{(k_3-\epsilon g_2)^2-4\epsilon k_3g_1}\right) . \end{aligned}$$

It is easily verified that $k_3>\epsilon _1^-g_2$ and this implies that $k_3+\lambda _4>0$ for $0<\epsilon <\epsilon _1^-$. Thus, if $0<\epsilon <\epsilon _1^-$, then the third and fourth components of the eigenvector $v_4$ have the same sign and the proof of Lemma B.1 again implies that rebound occurs.

The second part of the proof of Lemma B.1 considered the non-generic cases of the trajectory approaching the steady state tangent to one of the other eigenvectors. For the eigenvector $v_3$ in (59), we note that it is easily verified that $k_3+\lambda _3>0$ and so the same arguments apply in this case. The argument for the eigenvector $v_2$ is unchanged, while for $v_1$, the requirement for the third and fourth components of the eigenvector to be the same is $\epsilon g_2+\lambda _1<0$ and it is easily verified that this holds.

If $\epsilon =\epsilon _2$ corresponds to the point at which the eigenvalues $\lambda _1=-k_4$ and $\lambda _2$ coincide, then the conditions of Lemma B.1 hold with $\epsilon _0=\min (\epsilon _1^-,\epsilon _2)$ and so we conclude that rebound therefore occurs for all $\epsilon$ satisfying $0<\epsilon <\epsilon _0$. $\square$

This result shows that for this generalised model, rebound always occurs for sufficiently small $\epsilon >0$ even if there is no rebound without feedback ($\epsilon =0$), as is the case for the original TMDD model with feedback moderator.

Clearly, if the bottom right $2\times 2$ matrix in (68) has complex eigenvalues which have real part closest to zero of all the eigenvalues, then there will again be oscillation about the steady state in *y* and so rebound occurs infinitely often but with exponentially decreasing magnitude.

## Applications

We now consider two applications of these results to two different models. The first is the standard TMDD model with feedback. The second is a more complicated model of the effect of efalizumab on patients with psoriasis. This model is an application of the generalizations in Sect. 6.

### Example 1

We consider the TMDD equations including feedback with moderator given by (11)–(14). We use parameter values for the IgE mAb omalizumab (Sun 2001; Agoram et al. 2007), namely $k_\mathrm{e(L)}=0.024~\mathrm{day}^{-1}$, $k_\mathrm{e(P)}=0.201~\mathrm{day}^{-1}$, $k_\mathrm{out}=0.823~\mathrm{day}^{-1}$, $R_0=2.688~\mathrm{nM}$, $k_\mathrm{off}=0.900~\mathrm{day}^{-1}$, $k_\mathrm{on}=0.592~\mathrm{(nM\,day)}^{-1}$, $k_\mathrm{in}=k_\mathrm{out}R_0=2.212224~\mathrm{nM day}^{-1}$, and initial drug dose $L_0=14.8148$ nM. These parameter values have $k_\mathrm{e(L)}<k_\mathrm{e(P)}$ and so no rebound will occur when there is no feedback or in the case of direct feedback for any feedback function *H*(*R*). This follows directly from Theorem 2.1, Corollary 4.4, and Theorem 4.10 since $k_\mathrm{e(L)}<k_\mathrm{e(P)}$ implies that the parameter values are in the left green region in Figs. 3, 4, and 6.

However if there is feedback via a moderator then Theorem 5.12 implies that rebound occurs for all sufficiently small $\alpha >0$, even though there is no rebound at $\alpha =0$. The parameters for this example give

$$\begin{aligned} \lambda _1=-0.084, \quad \lambda _4=0.126, \quad k_3=0.517. \end{aligned}$$

**Fig. 10:**
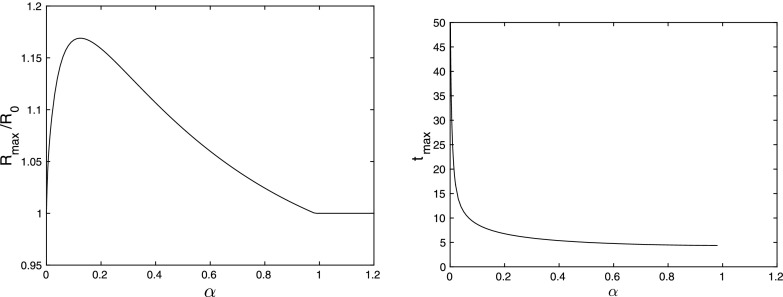
*Left* a plot of the relative magnitude of rebound $R_\mathrm{max}/R_0$ for feedback with a moderator and the mainly linear feedback function (34) with $H_0=1$ for a range of values of $\alpha$. *Right* a plot of the time at which the maximum rebound occurs

Thus Theorem 5.12 gives that the parameters are in the upper left region of Fig. 9 and that rebound will happen for $\alpha <0.135$ (using $\alpha =\epsilon /0.625$). However, this is a lower bound on the region of rebound. To illustrate this, we have used the mainly linear feedback function *H*(*R*) in (34) with $H_0=1$. The magnitude of the rebound is sufficiently small in this case that a nonlinear function $H_1(R)$ is not required. The maximum rebound is plotted in Fig. 10 for a range of values of $\alpha$ which does indeed show that there is rebound for all $\alpha >0$ and sufficiently small. For this example, the rebound ends at approximately $\alpha =0.98$. In this case, a local maximum for *R* persists for larger values of $\alpha$, but with $R_\mathrm{max}/R_0<1$. The value of time $t_\mathrm{max}$ at which the maximum rebound occurs is also shown in Fig. 10. We note that as $\alpha \rightarrow 0$ then $t_\mathrm{max}\rightarrow \infty$ and so the time of maximum rebound moves to infinity as the magnitude decreases. The same mechanism does not apply when the rebound disappears around $\alpha =1$ since a local maximum persists in this case but at values lower than baseline, as already discussed.

We have numerically sampled larger values of $\alpha$ and there is no indication of further rebound occurring. Of course we know that when $\alpha \rightarrow \infty$, the feedback with moderator becomes the direct feedback case, for which there is no rebound in this example.

### Example 2

In a study on the effect of efalizumab on patients with psoriasis (Ng et al. 2005), rebound was reported in some patients. The model equations proposed in Ng et al. (2005) are

$$\begin{aligned} \frac{dX_\mathrm{sc}}{dt}= & {} -k_aX_\mathrm{sc},\\ \frac{dX_1}{dt}= & {} -(k_{10}+k_{12})X_1+k_{21}X_2-\frac{V_\mathrm{m}X_1}{K_\mathrm{mc}V_\mathrm{c}+X_1} +F_\mathrm{a}k_\mathrm{a}X_\mathrm{sc},\\ \frac{dX_2}{dt}= & {} k_{12}X_1-k_{21}X_2,\\ \frac{dX_3}{dt}= & {} X_4-k_{30}X_3-\frac{V_\mathrm{m2}X_3X_1}{K_\mathrm{mc}V_\mathrm{c}+X_1},\\ \frac{dX_4}{dt}= & {} k_\mathrm{off}\left[ k_\mathrm{03max}\left( \frac{K_\mathrm{mc03}}{K_\mathrm{mc03}+X_3}\right) -X_4\right] , \end{aligned}$$

where $X_\mathrm{sc}$, $X_1$ and $X_2$ are the amounts of efalizumab in the depot, central and peripheral compartments respectively, $X_3$ is the total %CD11a on the surface of each T cell and $X_4$ is the production rate of %CD11a to the T cell surface. In this section we will show that this model can be written as a generalised model with feedback as studied in Sect. 6.1. Strictly speaking, this model is not a TMDD model as the efalizumab equations decouple from the %CD11a equations. As indicated in Ng et al. (2005), one could use $\frac{X_1X_3}{K_\mathrm{mc}V_\mathrm{c}+X_1}$ instead of $\frac{X_1}{K_\mathrm{mc}V_\mathrm{c}+X_1}$ in the PK equations and have a more TMDD-like model. In Ng et al. (2005), the simpler model was chosen as there was no difference in the fit of the data. If one would use the more complicated model, the parameters would need to be fitted again. So we focus in this section on the same model as in Ng et al. (2005). However, our analysis also applies to the more complicated model and the observed rebound would again be due to the slow feedback response.

In this model, $X_3$ is the total receptor, free and bound, and $X_4$ is the feedback moderator. The plots in Fig. 3 of Ng et al. (2005) show rebound in the total receptor, which of course is not the same as rebound in the free receptor. However, we first show that this model fits with the generalised model that we considered in Sect. 6.1 and we will then discuss the issue of rebound in the free receptor. To put this model into our framework, we take $\mathbf {X}=[X_\mathrm{sc},X_1,X_2]$, $Y=X_3$ and $F=X_4/k_\mathrm{03max}$. The differential equations for *Y* and *F* are then given by

70 $$\begin{aligned} \frac{dY}{dt}= & {} k_\mathrm{03max}F-k_{30}Y+F_2(\mathbf {X},Y), \end{aligned}$$

71 $$\begin{aligned} \frac{dF}{dt}= & {} k_\mathrm{off}\left( \frac{K_\mathrm{mc03}}{K_\mathrm{mc03}+Y}-F\right) , \end{aligned}$$

where

$$\begin{aligned} F_2(\mathbf {X},Y)=-\frac{V_\mathrm{m2}YX_1}{K_\mathrm{mc}V_\mathrm{c}+X_1}. \end{aligned}$$

The non-dimensional form of these equations is precisely in the form of Eqs. (63)–(65). These equations have the unique steady state in the region of interest given by

$$\begin{aligned} X_\mathrm{sc}^0=X_1^0=X_2^0=0,\quad Y^0=\frac{k_\mathrm{03max}F^0}{k_{30}}, \end{aligned}$$

where $F^0$ is the positive root of the quadratic equation

72 $$\begin{aligned} k_\mathrm{03max}F^2+k_{30}K_\mathrm{mc03}F-k_{30}K_\mathrm{mc03}=0. \end{aligned}$$

Substituting $F=1+\delta F$ into (72) gives a quadratic equation for $\delta F$ which has two negative roots, and this implies that $F^0\in (0,1)$. It is then easily verified that all the conditions stated in Sect. 6.1 on the functions $F_1$ and $F_2$ hold. The particular function *H* that is used in this model is given by

$$\begin{aligned} H(Y)=\frac{K_\mathrm{mc03}}{K_\mathrm{mc03}+Y}. \end{aligned}$$

We non-dimensionalise, considering only *Y* and *F*, by defining the new variables

$$\begin{aligned} y=\frac{Y}{Y^0},\quad w=\frac{F}{F^0},\quad \tau =V_\mathrm{m2}t. \end{aligned}$$

The non-dimensional form of equations (70)–(71) is then

$$\begin{aligned} \dot{y}= & {} k_3(w-y)+f_2(\mathbf {x},y),\\ \dot{w}= & {} \epsilon (h(y)-w), \end{aligned}$$

where dot denotes differentiation with respect to $\tau$, $\mathbf {x}$ is the (unspecified) non-dimensional form of $\mathbf {X}$, and

$$\begin{aligned} k_3=\frac{k_{30}}{V_\mathrm{m2}}, \quad \epsilon =\frac{k_\mathrm{off}}{V_\mathrm{m2}},\quad f_2(\mathbf {x},y)=\frac{F_2(\mathbf {X},Y^0y)}{V_\mathrm{m2}},\quad h(y)=\frac{1}{F^0+(1-F^0)y}. \end{aligned}$$

**Fig. 11:**
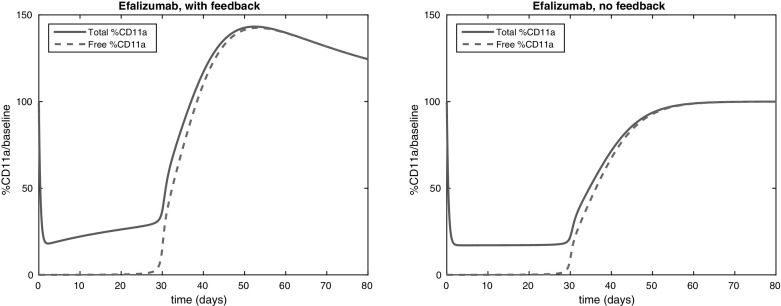
The total and free %CD11a relative to baseline after a single 3 mg/kg intravenous dose of efalizumab. On the *left* is the plot with feedback turned on and rebound can be observed. On the *right* is the plot without feedback and no rebound occurs

Note that we have used (72) in deriving *h*(*y*). All of our assumptions (h1)–(h4) on the function *h*(*y*) are now satisfied and

$$\begin{aligned} h_0=-h'(1)=1-F^0>0 \end{aligned}$$

since $F^0\in (0,1)$. We tried to reproduce the numerical results shown in Fig. 3B (dashed line) of Ng et al. (2005) but found that this was only possible by changing $k_\mathrm{off}$ from the stated value of 0.00154 day$^{-1}$ to 0.0154 day$^{-1}$ (the smaller value of $k_\mathrm{off}$ did show rebound, but with a smaller magnitude of about 110% of baseline). Moreover, the authors in Ng et al. (2005) report an affinity of 0.033 $\upmu$g/ml, which is $(0.033/150{,}000)\times 10^{-3}$M = $2 \times 10^{-10}$M, or 200pM. If we use the reported $k_\mathrm{off}$ of 0.00154 day$^{-1}$ and assume a typical $k_\mathrm{on}$ (see Aston et al. 2011) of 0.592 nM$^{-1}$/day, the ratio $k_\mathrm{off}/k_\mathrm{on}\approx 2$pM, i.e. 100-fold more potent than reported. A ten-times higher $k_\mathrm{off}$ still does not give the right affinity but at least it is closer. The values of the other parameters used, from Tables II and III of Ng et al. (2005), are $k_\mathrm{a}=0.242$ day$^{-1}$, $k_{10}=0.114$ day$^{-1}$, $k_{12}=0.097$ day$^{-1}$, $k_{21}=0.193$ day$^{-1}$, $k_{30}=0.444$ day$^{-1}$, $V_\mathrm{c}=64.3$ mL/kg, $V_\mathrm{m}=26.9~\upmu$g/mL, $V_\mathrm{m2}=2.16$ day$^{-1}$, $K_\mathrm{mc}=0.033~\upmu$g/mL, $F_\mathrm{a}=0.564$, $k_\mathrm{03max}=334$ %CD11a/day. The plot of the total receptor $X_3$ in this case is the solid blue line shown on the left in Fig. 11.

To find out how feedback influences the model, we ran a simulation of the model when the feedback was turned off, i.e., we took $X_4$ to be constant at its baseline value. As can be seen on the right in Fig. 11, without feedback there is no rebound. So clearly the rebound in this example is caused by the feedback.

Next we will interpret these results using our analysis. Using the parameter values in Ng et al. (2005), but the modified value of $k_\mathrm{off}$, we find that

$$\begin{aligned} \epsilon =7.13\times 10^{-3}, \end{aligned}$$

and since this is so small, we expect that the eigenvalue closest to zero of the Jacobian matrix evaluated at the steady state will come from the lower $2\times 2$ diagonal block. This is indeed the case since the eigenvalue closest to zero of this block is $-1.37\times 10^{-2}$ while the eigenvalue closest to zero from the remaining $3\times 3$ block $D_\mathbf {x}\mathbf {f}_{1}(0,1)$ is $-8.87\times 10^{-2}$. Thus, in the notation of Theorem 6.1, we have $0<\epsilon <\epsilon _0$ and so this Theorem says that we generically have rebound in the total receptor $X_3$, as confirmed in Fig. 11 and by the numerical results in Ng et al. (2005).

The two eigenvalues of the lower $2\times 2$ diagonal block collide at $\lambda =-0.122$ when $\epsilon =0.0390$, but since there are eigenvalues of $D_{\mathbf {x}}\mathbf {f}_{1}(0,1)$ that are closer to zero than this, we would not expect to see oscillatory convergence to the steady state in this model by simply increasing $\epsilon$ ($k_\mathrm{off}$), as described in Lemma B.3.

Finally, we recall that the variable $X_3$ in the above model is the total of the free and bound receptor. The free receptor on T cells is given by $K_\mathrm{mc}V_\mathrm{c}X_3/(K_\mathrm{mc}V_\mathrm{c}+X_1)$ (Ng et al. 2005) and we note that if $X_1=0$, then the free receptor is the same as the total receptor. Now $X_1$ is the amount of efalizumab in the central compartment and while this is present, the total receptor $X_3$ is kept at a low level. However, once $X_1$ has been almost depleted, then $X_3$ starts to rise again and rebound occurs. Thus, $X_1$ is very low in the phase of the dynamics in which rebound in $X_3$ occurs, and this implies that the free receptor is very similar to $X_3$ and so will also rebound. The free receptor for the above example is also shown in Fig. 11 as the red dashed line and it can clearly be seen that this also has rebound which is very similar to that for the total receptor as anticipated.

## Conclusions and discussion

In this paper we have extended our previous work on rebound in the basic TMDD model by including feedback. The feedback is negative, thus the rate of receptor production increases when the concentration drops below baseline (and vice versa). We modelled the feedback with an additional dynamic equation for the feedback moderator. If the feedback responds very fast, a quasi equilibrium approximation can be used and the feedback can be incorporated into the synthesis term itself.

We have shown that rebound is more likely to occur if feedback is present and that the likelihood of feedback occurring depends on the response speed of the feedback. If the feedback responds sufficiently slowly there will always be rebound. In the psoriasis example presented in Sect. 7.2, it can be seen that the rebound can be significant, an increase of over 40% of baseline is observed. The TMDD example in Sect. 7.1 shows that this is not always the case. In general, it is a challenge to describe the main parameters that influence the magnitude of the rebound. One important parameter is obviously the response speed of the feedback. If the response of the feedback goes to zero, then we converge to the case without feedback. Thus for many values of the elimination parameters the magnitude of the rebound will diminish if the response of the feedback slows down to zero and in the limit the rebound will have gone. The exceptions are of course those elimination parameter values for which rebound is observed if no feedback is present (the elimination rate of the antibody-protein complex is less than both the elimination rate of the antibody and the elimination rate of the protein ($k_\mathrm{e(P)}<k_\mathrm{e(L)}$ and $k_\mathrm{e(P)}<k_\mathrm{out}$)).

At first sight, it might be surprising that there is always rebound for a very slow feedback response rate ($0<\alpha \ll 1$), while there are large regions without rebound when the feedback moderator does not change and there is no feedback ($\alpha =0$). However, it can be seen that, in the absence of feedback dynamics ($\alpha =\epsilon =0$), a larger initial condition for the moderator *w* will lead to a steady state with $y=w$ which is above the baseline. Furthermore, Lemma 5.9 shows that when the moderator starts at baseline, ligand is added and feedback is turned on ($\alpha ,\epsilon >0$), then the moderator *w* initially increases to above its baseline value, albeit on a slow time scale since $\alpha$ is small. However, this implies that *w* will also only decrease back to its steady state value of 1 on a slow time scale. While $w>1$, *y* converges towards *w* on a faster time scale and will therefore itself exceed 1, resulting in rebound, followed by a slow relaxation of *y* and *w* back to their final steady state.

For moderately slow feedback response rates, the rebound is expected to be maximal, while for very fast feedback responses ($\alpha \gg 1$), the rebound will be approximated by the direct feedback limit. The results for the direct feedback model are qualitatively very similar to the results without feedback: the existence or non-existence of rebound again depends only on the three elimination rates $k_\mathrm{e(L)}$, $k_\mathrm{out}$ and $k_\mathrm{e(P)}$. An extra feature now is that the characteristics of the feedback function *H*(*R*) play a role as well if $k_\mathrm{e(L)}>k_\mathrm{e(P)}$. In particular, in both the direct feedback and no feedback models, there is no rebound if the elimination rate of the ligand is less than the elimination rate of the antibody-protein complex ($k_\mathrm{e(L)}<k_\mathrm{e(P)}$). Furthermore, in both models there will be rebound if the elimination rate of the antibody-protein complex is less than both the elimination rate of the antibody and the elimination rate of the protein ($k_\mathrm{e(P)}<k_\mathrm{e(L)}$ and $k_\mathrm{e(P)}<k_\mathrm{out}$). However, if $k_\mathrm{out}<k_\mathrm{e(P)}<k_\mathrm{e(L)}$, then the presence of feedback will increase the region in which rebound occurs. The details depend on the characteristics of the feedback function *H*(*R*) and can be found in Theorem 4.10. Again, in both models, the association and dissociation rate parameters $k_\mathrm{on}$ and $k_\mathrm{off}$ do not influence the occurrence of rebound, though they will play a role in the magnitude of the rebound if it occurs. There are some preliminary observations that a large $k_\mathrm{on}$ will dampen rebound, but this needs further work.

In the general feedback model, we have focused on proving the existence of rebound. In general, it is challenging to prove the non-existence of rebound as it has to be shown that the receptor is bounded by its baseline for all time. We expect that no rebound will occur for $k_\mathrm{e(L)}<k_\mathrm{e(P)}$ or small $k_\mathrm{out}$ for a sufficiently fast response rate ($\alpha$ large), but we have not yet obtained analytical results about this. The fast response is a singular limit and it can be shown that the dynamics has to be close to the limiting case. Thus if any rebound did happen, it would have to be very small.

Furthermore, we have also shown in this paper that most of the results obtained on rebound in the model with slow feedback can be extended to more general systems of TMDD equations such as equations with more compartments. We illustrated this with the example of psoriasis, where rebound is predicted by the model and underpins the observations in patients (see Sect. 7).

It must be acknowledged that during target discovery and validation, knowledge of the quantitative relationship between the target neutralisation and the nature of the negative feedback is often not known. However, using prior knowledge on the target of interest and similar targets, the model can be used to evaluate the possibility of feedback and its potential magnitude, and thus confirm the suitability of the target.

## Figures and Tables

**Fig. 12:**
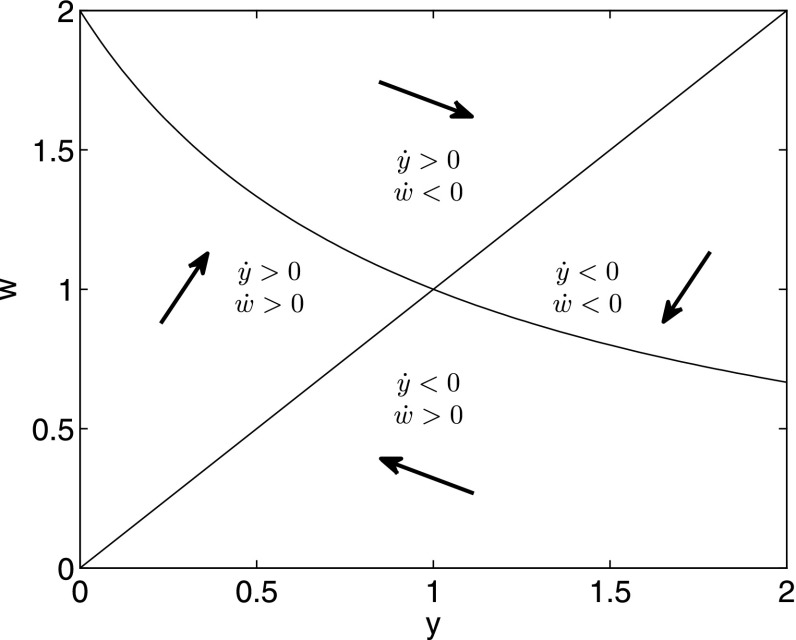
The two nullclines for Eqs. (49), (50) divide the phase plane into four regions in which $\dot{y}$ and $\dot{w}$ have one sign

**Fig. 13:**
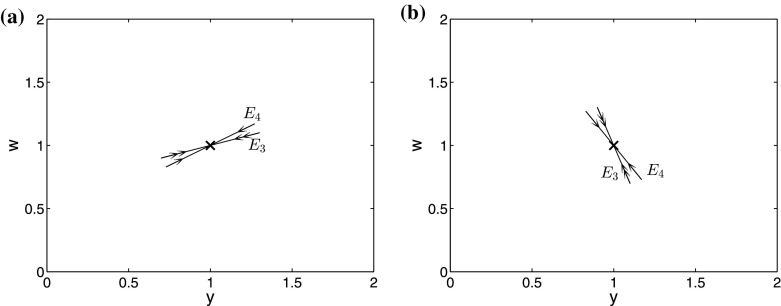
The linearised manifolds $E_{3,4}$ associated with the eigenvalues $\lambda _{3,4}$ **a** $\epsilon <\epsilon _1^-$, **b** $\epsilon >\epsilon _1^+$

## Appendix A Proofs of invariance and stability results

### Proof of Lemma 4.1

The phase space is bounded by the three planes $x=0$, $y=0$ and $z=0$. If the vector field given by the Eqs. (16)–(18) on these planes is directed inside or is tangent to the octant, then the given region is invariant (Smith 1995).

Equations (16) and (17) are the same as in the simple TMDD model, and so the direction of the vector field on the planes $x=0$ and $z=0$ was established in the proof of Lemma 2.1 of Aston et al. (2014) (see also Peletier and Gabrielsson 2012).

The only difference in this case is in the derivative normal to the plane $y=0$, which is now

$$\begin{aligned} \dot{y}|_{y=0}=k_3h(0)+k_2z. \end{aligned}$$

From assumption (h3), we see that $h(0)>1$ and so $\dot{y}|_{y=0}>0$ when $z\ge 0$ as before. The rest of the proof is unchanged. $\square$

### Proof of Lemma 4.2

Setting the derivatives to zero in (16) and (17) and solving for *x* and *z*, the only valid solution possible is $x=z=0$. Substituting these into (18) with the derivative set to zero, we obtain

$$\begin{aligned} k_3(h(y)-y)=0. \end{aligned}$$

By assumptions (h2) and (h3), $y=1$ is the unique non-negative solution of this equation. Thus the unique steady state in the positive octant of Eqs. (16)–(18) is given by $x=z=0$ and $y=1$.

To show the global stability, we note that $u=x+\mu z$ again decreases monotonically to zero using the same approach as in Lemma 2.3 of Aston et al. (2014) and since *x* and *z* must be non-negative, we conclude that $\lim _{\tau \rightarrow \infty }x(\tau )=\lim _{\tau \rightarrow \infty }z(\tau )=0$, as for the original TMDD model. Similar to the original TMDD model, the *y*-axis, i.e, the manifold $x=0$, $z=0$, forms an invariant manifold as $\dot{x}\mid _{x=0,\, z=0}=0$ and $\dot{z}\mid _{x=0,\, z=0}=0$. On this invariant manifold, the *y*-dynamics is given by

$$\begin{aligned} \dot{y} = k_3(h(y)-y) . \end{aligned}$$

From the assumptions on the function *h*, it follows immediately that for $0<y<1$, $h(y)-y>1-y>0$; for $y>1$, $h(y)-y<1-y<0$; and for $y=1$, we have $h(y)-y=1-1=0$. Thus $y=1$ is an attractor for the positive *y*-axis. Intuitively, these two observations together give that the steady state $(x,y,z)=(0,1,0)$ is a global attractor for the positive octant. To make this formal, we consider the *y*-dynamics in a neighbourhood of the *y*-axis.

Let $\epsilon >0$. Since $\lim _{\tau \rightarrow \infty }x(\tau )= \lim _{\tau \rightarrow \infty }z(\tau )=0$, then there is some $T>0$ such that for $\tau >T$, we have

$$\begin{aligned} 0\le x(\tau )\le \epsilon \mu k_3 \quad \hbox {and} \quad 0\le z(\tau )\le \epsilon k_3/k_2. \end{aligned}$$

Thus for $\tau >T$, the *y*-dynamics (18) can be estimated by

$$\begin{aligned} k_3 \left[ h(y) - (1+\epsilon ) y)\right] \le \dot{y} \le k_3 \left[ h(y) + \epsilon - y \right] . \end{aligned}$$

If $y(T)>1$, then $y(\tau )\ge 1$ on some $\tau$-interval beyond *T*. In this interval we have $h(y(\tau ))\le 1$, thus

$$\begin{aligned} \dot{y} \le k_3 \left[ 1 + \epsilon - y \right] \quad \hbox {or}\quad \frac{d}{d\tau } \left[ e^{k_3\tau } \left( y-(1+\epsilon )\right) \right] \le 0. \end{aligned}$$

Thus

73 $$\begin{aligned} y(\tau ) \le (1+\epsilon ) + e^{-k_3(\tau -T)} \left( y(T)-(1+\epsilon )\right) . \end{aligned}$$

Similarly, if $y(T)<1$, then $y(\tau )\le 1$ on some $\tau$-interval beyond *T*. In this interval we have $h(y(\tau ))\ge 1$, thus

$$\begin{aligned} \dot{y} \ge k_3 \left[ 1 - (1+\epsilon ) y)\right] \quad \hbox {or}\quad \frac{d}{d\tau } \left[ e^{k_3(1+\epsilon )\tau }\left( y-\frac{1}{1+\epsilon }\right) \right] \ge 0 \end{aligned}$$

Thus

74 $$\begin{aligned} y(\tau ) \ge 1-\frac{\epsilon }{1+\epsilon } + e^{-k_3(1+\epsilon )(\tau -T)}\left( y(T)-\frac{1}{1+\epsilon }\right) . \end{aligned}$$

If $y(\tau )> 1$ for all $\tau \ge T$, then the estimate (73) is valid for all $\tau >T$ and we can conclude that there is some $T_1>0$ such that $1\le y(\tau ) \le 1+2\epsilon$ for $\tau >T_1$. Similarly, if $y(\tau )< 1$ for all $\tau \ge T$, then the estimate (74) is valid for all $\tau >T$ and we can again conclude that there is some $T_1>0$ such that $1\ge y(\tau ) \ge 1-2\epsilon$ for $\tau >T_1$

However, it is also possible that there is some $\widehat{T}\ge T$ such that $y(\widehat{T})=1$. In this case, (73) gives that if $y(\tau )\ge 1$ in some interval $[\widehat{T},\widehat{T}_1]$, then we can estimate for $\tau$ in this interval

$$\begin{aligned} 1\le & {} y(\tau ) \le (1+\epsilon ) + e^{-k_3(\tau -T)} \left( y(\widehat{T})-(1+\epsilon )\right) \\= & {} (1+\epsilon ) + e^{-k_3(\tau -T)} \left( 1-(1+\epsilon )\right) \le (1+\epsilon ) \end{aligned}$$

Similarly, if $y(\tau )\le 1$ in some interval $[\widehat{T},\widehat{T}_1]$, then (74) gives that for $\tau$ in this interval, we have

$$\begin{aligned} 1\ge & {} y(\tau ) \ge 1-\frac{\epsilon }{1+\epsilon } + e^{-k_3(1+\epsilon )(\tau -T)}\left( y(\widehat{T})-\frac{1}{1+\epsilon }\right) \\= & {} 1-\frac{\epsilon }{1+\epsilon } + e^{-k_3(1+\epsilon )(\tau -T)}\left( 1-\frac{1}{1+\epsilon }\right) \ge 1-\epsilon \end{aligned}$$

Thus if there is some $\widehat{T}\ge T$ such that $y(\widehat{T})=1$, then the interval $[\widehat{T},\infty )$ can be divided into intervals [*a*, *b*) such that $y(a)=1=y(b)$ and $y(\tau )-1$ is either negative or positive for $\tau \in (a,b)$. On each of those intervals, one of the two estimates above can be used and hence we get that $|y(\tau )-1|<\epsilon <2\epsilon$ for all $\tau \ge \widehat{T}$.

Combining these three cases, we can conclude that for all $\epsilon >0$, there is some $\widetilde{T}>0$ such that $|y(\tau )-1|<\epsilon <2\epsilon$ for all $\tau >\widetilde{T}$. Thus $\lim _{\tau \rightarrow \infty } y(\tau )=0$ and we can conclude that all trajectories must converge to the steady state $(x,y,z)=(0,1,0)$ as $t\rightarrow \infty$. $\square$

### Proof of Lemma 5.2

From (53) we note that

$$\begin{aligned} \lambda _4= & {} \frac{1}{2}(k_3+\epsilon )\left( -1+\sqrt{1-\frac{4\epsilon k_3(1+h_0)}{(k_3+\epsilon )^2}}\right) = \frac{1}{2}(k_3+\epsilon )\left( \frac{\frac{4\epsilon k_3(1+h_0)}{(k_3+\epsilon )^2}}{-1-\sqrt{1-\frac{4\epsilon k_3(1+h_0)}{(k_3+\epsilon )^2}}}\right) \\= & {} \frac{\frac{2\epsilon k_3(1+h_0)}{(k_3+\epsilon )}}{-1-\sqrt{1-\frac{4\epsilon k_3(1+h_0)}{(k_3+\epsilon )^2}}} \end{aligned}$$

Taking the limit in this expression as $\epsilon \rightarrow \infty$ gives the stated result. $\square$

### Proof of Lemma 5.3

We define the Lyapunov function

$$\begin{aligned} V(y,w)=\frac{1}{k_3}\int _1^y (1-h(y'))~dy'+\frac{1}{2\epsilon }(w-1)^2. \end{aligned}$$

For the steady state to be globally asymptotically stable, we have to prove that *V* is positive definite and continuous and that $\dot{V}$ is negative definite and continuous relative to the steady state $y=w=1$ on the whole of the phase space (Jordan and Smith 2007).

By hypothesis (h4), *V* and $\dot{V}$ are continuous. To show that *V* is positive definite, we note that $V(1,1)=0$ and that hypothesis (h3) implies that

75 $$\begin{aligned} 1-h(y)\quad \left\{ \begin{array}{ll}<0 &{}\quad \hbox {if}~y<1\\ =0 &{}\quad \hbox {if}~y=1\\>0 &{}\quad \hbox {if}~y>1 \end{array}\right. \end{aligned}$$

Thus,

$$\begin{aligned} \int _1^y (1-h(y'))~dy'\quad \left\{ \begin{array}{ll} >0 &{} \quad \hbox {if}~y\ne 1\\ =0 &{}\quad \hbox {if}~y=1 \end{array}\right. \end{aligned}$$

and so $V(y,w)>0$ if $(y,w)\ne (1,1)$.

Differentiating *V* with respect to $\tau$ gives

$$\begin{aligned} \dot{V}= & {} \frac{1}{k_3}(1-h(y))\dot{y}+\frac{1}{\epsilon }(w-1)\dot{w}\\= & {} (1-h(y))(w-y)+(w-1)(h(y)-w)\\= & {} -(w-1)^2-(1-h(y))(y-1). \end{aligned}$$

We note that (75) implies that $(1-h(y))(y-1)>0$ if $y\ne 1$ and so $\dot{V}<0$ if $(y,w)\ne (1,1)$. Also, $\dot{V}(1,1)=0$. Thus, $\dot{V}$ is negative definite.

As all the above conditions are satisfied, we conclude that the steady state $(y,w)=(1,1)$ is globally asymptotically stable. $\square$

### Proof of Lemma 5.4

We first observe that the nullclines for Eqs. (49), (50) are given by the two curves

$$\begin{aligned} w=y,\quad w=h(y) \end{aligned}$$

which intersect at the steady state $y=w=1$. These two curves divide the phase plane into four regions in which the signs of $\dot{y}$ and $\dot{w}$ do not change, as illustrated in Fig. 12.

We note from (55) that

$$\begin{aligned} k_3-\epsilon _1^\pm= & {} k_3\left( -2h_0\mp \sqrt{4h_0(1+h_0)}\right) \\= & {} -2k_3h_0\left( 1\pm \sqrt{1+\frac{1}{h_0}}\right) \end{aligned}$$

and it follows from this that

76 $$\begin{aligned} \epsilon _1^-<k_3<\epsilon _1^+. \end{aligned}$$

We next observe from (52) and (53) that

$$\begin{aligned} \lambda _i+k_3=\frac{1}{2}\left( k_3-\epsilon \pm \sqrt{(k_3-\epsilon )^2-4\epsilon k_3h_0} \right) , \quad i=3,4 \end{aligned}$$

and it is easily verified from this that

77 $$\begin{aligned} \lambda _i+k_3~\left\{ \begin{array}{ll}<0&{}\quad \mathrm{if}~\epsilon>k_3,\\ >0&{}\quad \mathrm{if}~\epsilon <k_3, \end{array}\right. \quad i=3,4. \end{aligned}$$

We now consider the two eigenvectors associated with the eigenvalues $\lambda _3$ and $\lambda _4$, which are given by (54). The slopes of the linearised manifolds $E_{3,4}$ associated with the eigenvectors $v_{3,4}$ in the (*y*, *w*) plane are $(k_3+\lambda _i)/k_3$, $i=3,4$. We now consider separately the two ranges in $\epsilon$ for which the eigenvalues are real.

If $0<\epsilon <\epsilon _1^-$, then from (76), we also have that $\epsilon <k_3$. From (77), this implies that $\lambda _i+k_3>0$, $i=3,4$ and so the slopes of $E_3$ and $E_4$ are both positive and as $\lambda _{3,4}<0$, the slopes are also both less than one. Since $\lambda _3<\lambda _4$, the manifold associated with $\lambda _4$ has the steepest slope. This situation is shown in Fig. 13a.

Since $\lambda _4$ is the eigenvalue closest to zero, a generic trajectory will approach the steady state tangent to the linearised manifold $E_4$. Now the global one-dimensional stable manifolds which are tangent to $E_3$ and $E_4$ at the steady state are invariant, and hence no trajectory can cross them. Thus, all initial conditions with $y(0)<1$ but above the global stable manifold tangent to $E_3$ will rotate round the fixed point in a clockwise direction, as indicated in Fig. 12, and will approach the steady state tangent to $E_4$ from the right of the steady state, for which $y>1$. Now this global manifold must itself spiral in to the steady state in a clockwise direction as shown in Fig. 12 and so cannot cross the line $w=1$, $0\le y\le 1$, at least not unless it circles the steady state first. Thus, all initial conditions satisfying $w(0)>1$, $y(0)<1$ will give rise to trajectories with rebound as they approach the steady state, as will initial conditions with $w(0)<1$, $y(0)<1$ that lie above the global manifold that is tangent to $E_3$ at the steady state.

When $\epsilon >\epsilon _1^+$, an analogous process can be followed to show that $E_3$ and $E_4$ both have negative slopes which are less than $h'(1)$ and this is shown in Fig. 13b. Again, any initial conditions to the right of the global manifold tangent to $E_3$ will rotate around the steady state in a clockwise direction and will approach the steady state with $y>1$, giving rebound. As $\epsilon \rightarrow \infty$, we have already seen that $\lambda _3\rightarrow -\infty$, and in this case, $E_3$ becomes vertical and so the region of initial conditions that gives rise to rebound, near to the steady state at least, becomes smaller as $\epsilon$ increases. Thus, for all $\epsilon \in (\epsilon _1^+,\infty )$, there are always some initial conditions with $w(0)>1$, $y(0)<1$ which will give rise to rebound, as claimed.

We note that as the global stable manifolds may spiral in to the steady state in a clockwise direction, we cannot say whether initial conditions satisfying $w(0)<1$, $y(0)<1$ are above or below this global manifold, and so we cannot comment on the existence or non-existence of rebound in this case.

When $\epsilon =\epsilon _1^\pm$, the eigenvalues $\lambda _3$ and $\lambda _4$ collide as well as their eigenvectors. Instead of two eigenvectors, there is now one eigenvector $v_3=v_4$ and a generalised eigenvector. All solutions will approach the steady state via the eigenvector, see Jordan and Smith (2007). The analysis for the approach to the steady state for the case $0<\epsilon <\epsilon _1^-$ can be extended to the case $\epsilon =\epsilon _1^-$ and similarly, the case $\epsilon >\epsilon _1^+$ can be extended to $\epsilon =\epsilon _1^+$.

The last case to consider are the complex eigenvalues when $\epsilon _1^-<\epsilon <\epsilon _1^+$. When $\epsilon$ is in this range, the eigenvalues $\lambda _{3,4}$ are a complex conjugate pair. Thus close to the steady state, trajectories will spiral around it. Each time the trajectory loops round the steady state, there will be a part of the trajectory with $y>1$ and so rebound occurs infinitely often, but with exponentially decreasing magnitude, which is determined by $\mathrm{Re}(\lambda _{3,4})= -(k_3+\epsilon )/2$. $\square$

### Proof of Lemma 5.5

The phase space is bounded by the planes $x=0$, $y=0$, $z=0$ and $w=0$. As in the proof of Lemma 4.1, we have to show that the derivative normal to each of these planes is non-negative (Smith 1995).

The proof that the derivative normal to the planes $x=0$ and $z=0$ is non-negative is the same as in the proof of Lemma 4.1. On the plane $y=0$, we find from (22) that

$$\begin{aligned} \dot{y}|_{y=0}=k_3w+k_2z \end{aligned}$$

and so $\dot{y}|_{y=0}\ge 0$ when $w,z\ge 0$. Similarly on the plane $w=0$, from (23), we obtain

$$\begin{aligned} \dot{w}|_{w=0}=\epsilon h(y) \end{aligned}$$

and so $\dot{w}|_{w=0}>0$ for all $y\ge 0$ using assumption (h1).

Thus, we conclude that any trajectory with *x*(0), *y*(0), *z*(0), $w(0)\ge 0$ must satisfy $x(\tau )$, $y(\tau )$, $z(\tau )$, $w(\tau )\ge 0$ for all $\tau >0$. $\square$

### Proof of Lemma 5.6

Solving the equations $\dot{x}=\dot{y}=\dot{z}=0$ for *x*, *y* and *z* again gives two solutions, but one of these has *y* negative, so is not physically relevant and is not in the positive part of the phase plane considered in this lemma. The other solution is given by

$$\begin{aligned} x=z=0,\quad y=w. \end{aligned}$$

Substituting for *y* into the equation $\dot{w}=0$ gives

78 $$\begin{aligned} h(w)-w=0. \end{aligned}$$

As in the proof of Lemma 4.2, we see that $w=1$ is a solution of this equation using (h2) and assumption (h3) ensures that this solution is unique. $\square$

### Proof of Lemma 5.7

We showed in Aston et al. (2014) that $\lambda _1$ and $\lambda _2$ are always real and negative. Furthermore, Lemma 5.2 gives that $\lambda _3$ and $\lambda _4$ are always negative and real or complex with negative real part. So the steady state is linearly stable. $\square$

### Proof of Lemma 5.8

The linear stability proved in Lemma 5.7 implies local stability, i.e., there is some $\delta >0$ such that all solutions in a $\delta$ neighbourhood of the fixed point (0, 0, 1, 1) will converge to this fixed point as $\tau \rightarrow \infty$. Or formally, if at some $\tau _0$ we have $||(x(\tau _0),z(\tau _0),y(\tau _0),w(\tau _0))-(0,0,1,1)||<\delta$, then $\lim _{\tau \rightarrow \infty } (x(\tau ),z(\tau ),y(\tau ),w(\tau ))=(0,0,1,1)$. For later use, we define the following quantities

$$\begin{aligned} b_1= & {} \min \left\{ h(y)-1\mid 0\le y \le 1-\frac{\delta }{2}\right\} , \quad b_2 = \min \left\{ 1-h(y)\mid y \ge 1+\frac{\delta }{2}\right\} ,\\ b= & {} \min (b_1,b_2); \end{aligned}$$

and

$$\begin{aligned} B= \sup \{|h(y)-1|\mid y\ge 0\}. \end{aligned}$$

The assumptions (h1)–(h4) ensure that $b>0$ and $|h(y)-1|$ is bounded, hence *B* is well defined.

Let $(x(\tau ),z(\tau ),y(\tau ),w(\tau ))$ be a solution of the TMDD system with moderator feedback (20)–(23). Following the same arguments as in Lemma 2.3 of Aston et al. (2014), it follows that the normalised total amount of drug $u=x+\mu z$ decreases monotonically to zero. Since *x* and *z* must be non-negative, this implies that $\lim _{\tau \rightarrow \infty }x(\tau )=\lim _{\tau \rightarrow \infty }z(\tau )=0$, as for the original TMDD model. Thus there is some $T>0$ such that for all $\tau >T$, we have

79 $$\begin{aligned} |x(\tau )|< \min \left( \frac{b\delta }{8},\frac{\delta ^2}{16}\right) \min \left( \frac{\mu k_3}{B}, 1\right) \hbox { and } |z(\tau )| < \min \left( \frac{b\delta }{8},\frac{\delta ^2}{16}\right) \min \left( \frac{k_3}{k_2B}, 1\right) .\nonumber \\ \end{aligned}$$

Next we consider the function *V*(*y*, *w*) as defined in the proof of Lemma 5.3, i.e.,

$$\begin{aligned} V(y,w)=\frac{1}{k_3}\int _1^y (1-h(y'))~dy'+\frac{1}{2\epsilon }(w-1)^2. \end{aligned}$$

It is shown in that proof that *V* is non-negative for $y\ge 0$ and $w\ge 0$. We will show here that $V(y(\tau ),w(\tau ))$ is decreasing when $\tau >T$ and $|y(\tau )-1|\ge \frac{\delta }{2}$ or $|w(\tau )-1|\ge \frac{\delta }{2}$ and that this implies that any solution has to enter the local $\delta$ neighbourhood in which solutions get attracted to the fixed point.

Let $\tau >T$, i.e, $x(\tau )$ and $z(\tau )$ satisfy (79). Then we have

$$\begin{aligned} \dot{V}(y,w)= & {} \frac{1}{k_3}\,(1-h(y))\,\dot{y}+\frac{(w-1)\dot{w}}{\epsilon }\\= & {} \frac{1}{k_3}\,(1-h(y))\,\left( k_3(w-y)-\frac{xy}{\mu }+ k_2 z\right) + (w-1) (h(y)-w)\\= & {} -(w-1)^2-(1-h(y))(y-1) - \frac{(1-h(y))}{k_3}\left( \frac{xy}{\mu }- k_2 z\right) . \end{aligned}$$

Next we consider each term. First we note that for every $w\ge 0$ and $y\ge 0$, we have that $-(w-1)^2\le 0$ and $-(1-h(y))(y-1) \le 0$. Furthermore, if $|w-1|\ge \frac{\delta }{2}$, then $-(w-1)^2\le -\frac{\delta ^2}{4}$. Also, if $|y-1|\ge \frac{\delta }{2}$ then the fact that $(1-y)(h(y)-1)>0$ and the definition of *b* give that $-(1-y)(h(y)-1)\le -\frac{b\delta }{2}$. The definition of *B* gives that $\frac{(1-h(y))k_2z}{k_3}\le \frac{Bk_2|z|}{k_3}< \min \left( \frac{b\delta }{8},\frac{\delta ^2}{16}\right)$. Finally, if $y>1$, then $1-h(y)>0$, thus $-\frac{(1-h(y))}{k_3}\frac{xy}{\mu }\le 0$. If $0\le y\le 1$, then the definition of *B* gives $-\frac{(1-h(y))}{k_3}\frac{xy}{\mu }\le \frac{B}{k_3}\frac{x}{\mu }$. So together we get that $-\frac{(1-h(y))}{k_3}\frac{xy}{\mu }< \min \left( \frac{b\delta }{8},\frac{\delta ^2}{16}\right)$. With these four estimates we get that if $|w-1|\ge \frac{\delta }{2}$ or $|y-1|\ge \frac{\delta }{2}$, then

$$\begin{aligned} \dot{V}(y(\tau ),w(\tau )) < -\min \left( \frac{b\delta }{2},\frac{\delta ^2}{4}\right) + 2 \min \left( \frac{b\delta }{8},\frac{\delta ^2}{16}\right) = - \min \left( \frac{b\delta }{4},\frac{\delta ^2}{8}\right) . \end{aligned}$$

If we assume that for all $\tau >T$, either $|w(\tau )-1|\ge \frac{\delta }{2}$ or $|y(\tau )-1|\ge \frac{\delta }{2}$ (or both), then integration of the estimate above gives that

$$\begin{aligned} V(\tau )< V(T) - \min \left( \frac{b\delta }{4},\frac{\delta ^2}{8}\right) (\tau -T)<0 \end{aligned}$$

for $\tau$ sufficiently large. However this contradicts the fact that *V* is non-negative, hence our assumption is incorrect and we can conclude that there is some $\tau _0>T$ such that $|w(\tau _0)-1|<\frac{\delta }{2}$ and $|y(\tau _0)-1|<\frac{\delta }{2}$. Since $\tau _0>T$, we also have $|x(\tau _0)|<\frac{\delta ^2}{16}$ and $|z(\tau _0)|<\frac{\delta ^2}{16}$. Thus $||(x(\tau _0),z(\tau _0),y(\tau _0),w(\tau _0))-(0,0,1,1)||<\delta$ and the local stability implies that the solution will converge to the steady state as $\tau \rightarrow \infty$. $\square$

## Appendix B Proof of rebound lemmas

Lemma 5.13 is proved by assuming that $\lambda _4$ is closest to zero and then considering the three $\epsilon$ intervals for which the value of $\lambda _4$ is qualitatively different.

### Lemma B.1

If $0<\epsilon \le \epsilon _1^-$ and the eigenvalue $\lambda _4$ is closest to zero then rebound occurs.

### Proof

First we consider the case $0<\epsilon <\epsilon _1^-$. Generically the trajectory must approach the steady state tangent to the eigenvector $v_4$ given in (59) since $\lambda _4$ is real and is the eigenvalue closest to zero. We note that

$$\begin{aligned} k_3+\lambda _4=\frac{1}{2}\left( k_3-\epsilon + \sqrt{(k_3-\epsilon )^2-4\epsilon k_3h_0}\right) . \end{aligned}$$

We see from (53) that the term under the square root is positive if $\lambda _4$ is real (i.e., $\epsilon \not \in (\epsilon _1^-,\epsilon _1^+)$), and thus the expression above immediately implies that $k_3+\lambda _4>0$ if $\epsilon<\epsilon _1^-<k_3$ and $k_3+\lambda _4<0$ if $\epsilon>\epsilon _1^+>k_3$. Therefore, if $0<\epsilon <\epsilon _1^-$, then the third and fourth components of the eigenvector $v_4$ have the same sign. We therefore have two cases to consider. In the first case, *y* and *w* both approach the steady state from above, and clearly rebound occurs in this case. Alternatively, *y* and *w* both approach the steady state from below and Corollary 5.11 implies that there is rebound in this case also.

As in the proof of Theorem 3.5 in Aston et al. (2014), we must again consider the possibility that for particular parameter values, the generic situation considered above does not occur, and so the trajectory approaches the steady state tangent to one of the other eigenvectors.

We first consider the eigenvector $v_3$ (see (59)). For this vector, we note that $k_3+\lambda _3>0$, by a similar argument to the one above for $k_3+\lambda _4$, and so the third and fourth components of the eigenvector $v_3$ have the same sign. Thus, the above proof by contradiction also holds if the trajectory approaches the steady state tangent to $v_3$.

For the eigenvector $v_2$, we note, as in the proof of Theorem 3.5 of Aston et al. (2014), that this eigenvector points out of the phase space, since the first and second components have opposite sign, and so no trajectory can approach the steady state tangent to this eigenvector.

Finally we consider the eigenvector $v_1$. We note that the third and fourth components of this eigenvector will have the same sign if $\epsilon +\lambda _1<0$. Since $\lambda _4$ is real and closest to zero, the definition of $\lambda _4$ (53) gives

$$\begin{aligned} \epsilon +\lambda _1<\epsilon +\lambda _4 = \frac{1}{2}\left( \epsilon -k_3+ \sqrt{(k_3-\epsilon )^2-4\epsilon k_3h_0}\right) < 0 \end{aligned}$$

as $\epsilon<\epsilon _1^-<k_3$. So we can conclude that the third and fourth entries in the eigenvector $v_1$ have the same sign, and so the above proof by contradiction also holds if the trajectory approaches the steady state tangent to $v_1$.

This covers all possible cases for $\epsilon <\epsilon _1^-$, and so we conclude that rebound must occur for those $\epsilon$ values.

Finally we consider the case $\epsilon =\epsilon _1^-$, which is when the Jacobian matrix has two repeated real eigenvalues $\lambda _3=\lambda _4(=\lambda _*^-)$ that are the least negative. In this case, the two corresponding eigenvectors satisfy $v_3=v_4$ and there is a generalised eigenvector $v_g$ which is given by

$$\begin{aligned} v_g=(0,0,0,1) \end{aligned}$$

The solutions on the two-dimensional linearised manifold spanned by $v_3$ and $v_g$ are linear combinations of $e^{\lambda _3 t}(v_g+tv_3)$ and $e^{\lambda _3 t}v_3$ (Jordan and Smith 2007) and asymptotically will align with the eigenvector $v_3$. It is easily verified that $k_3+\lambda _*^->0$, and so the third and fourth components of $v_3$ have the same sign. The same arguments as above show that rebound must then occur in this case, and that the trajectory cannot approach the steady state tangent to $v_1$ or $v_2$. $\square$

Next we consider the case that $\epsilon >\epsilon _1^+(k_3)$ and $\lambda _4$ is real and the eigenvalue closest to zero. This can only happen if $\lambda _1<\lambda _\infty$. In the case of the direct feedback, we analysed this region by using that the total amount of receptor ($v=y+z$) stays on one side of the plane $v=1$, see Lemma 4.7. As observed earlier, this property does not hold any more for the feedback with a moderator, but we can define a related quantity and show a partial result.

We write *h*(*y*) as in (35), i.e., $h(y)=1+(1-y)\tilde{h}(y)$ and define the bounds *m* and *M* as in (41). Next we define

$$\begin{aligned} \tilde{v} = y+z+ \frac{k_3\,w}{\epsilon }. \end{aligned}$$

At baseline, we have $\tilde{v} = 1+\frac{k_3}{\epsilon }$ and the initial condition is also in the plane $\tilde{v} = 1+\frac{k_3}{\epsilon }$. By using this plane, we will now prove that there is rebound if $k_3$ is sufficiently large compared to ${k_4}$.

### Lemma B.2

If $\epsilon \ge \epsilon _1^+$, the eigenvalue $\lambda _4$ is closest to zero, and $k_3>\frac{k_4}{1+m}$, then rebound occurs.

### Proof

Using the definition of $\tilde{v}$, we get that

80 $$\begin{aligned} \dot{\tilde{v}}= & {} -k_4z + k_3(h(y)-y) \nonumber \\= & {} (1-y)(k_3(1+\tilde{h}(y))-k_4) + k_4\left( 1+\frac{k_3}{\epsilon }-\tilde{v}\right) + \frac{k_3k_4}{\epsilon }(w-1). \end{aligned}$$

Evaluating this expression at $\tau =0$, gives $\dot{\tilde{v}}(0)=0$. Differentiating once more shows that

$$\begin{aligned} \ddot{\tilde{v}} (0) = \frac{1}{\mu }\,(k_3(1+h_0)-k_4)>0, \end{aligned}$$

as $k_4<k_3(1+m)<k_3(1+h_0)$. Thus $\tilde{v}(\tau )>1+\frac{k_3}{\epsilon }$ for sufficiently small $\tau >0$.

We will prove the occurrence of rebound with a contradiction argument and so we assume that there is no rebound, i.e., $y(\tau )<1$ for all $\tau >0$. With Lemma 5.10, this implies that $w(\tau )>1$ for all $\tau >0$. Hence on the plane $\tilde{v} =1+\frac{k_3}{\epsilon }$, Eq. (80) gives

$$\begin{aligned} \dot{\tilde{v}}|_{\tilde{v} =1+\frac{k_3}{\epsilon }} = (1-y)(k_3(1+\tilde{h}(y))-k_4) + \frac{k_3k_4}{\epsilon }(w-1) > (1-y)(k_3(1+m)-k_4) . \end{aligned}$$

Since $k_3(1+m)>k_4$, this implies that $\dot{\tilde{v}}|_{\tilde{v} \!=\! 1+\frac{k_3}{\epsilon }} >0$, hence $\tilde{v}(\tau )> 1+\frac{k_3}{\epsilon }$ for all $\tau >0$.

Since $\lambda _4$ is the eigenvalue closest to zero, generically the solution will approach the steady state along the line spanned by the eigenvector $v_4$. Furthermore, the outward normal to the plane $\tilde{v} =1+\frac{k_3}{\epsilon }$ is $\nabla \tilde{v}=(0,1,1,k_3/\epsilon )$. Thus with the expression for $v_4$ in (59) and $\epsilon >\epsilon ^+_1$, it follows that its fourth component is

$$\begin{aligned} k_3+\lambda _4< k_3+\lambda _\infty = k_3-k_3(1+h_0) = -k_3h_0<0. \end{aligned}$$

As the solution has $y<1$ and $w>1$, the solution comes in via $-v_4$. But this vector is below the plane $\tilde{v} =1+\frac{k_3}{\epsilon }$ as

$$\begin{aligned} (\nabla \tilde{v},-v_4) = -\frac{k_3}{\epsilon } \,(\epsilon +k_3+\lambda _4) < 0, \end{aligned}$$

as, by the definition of $\lambda _4$, we have $\epsilon +k_3+\lambda _4 = \frac{1}{2}(k_3+\epsilon +\sqrt{D})>0$. This contradicts the fact that the solution is always above the plane, hence our assumption that there is no rebound is false.

Finally, we consider the case $\epsilon =\epsilon _1^+$. This case is similar to that when $\epsilon =\epsilon _1^-$ in the proof of Lemma B.1 in that there is a repeated eigenvalue $\lambda _3=\lambda _4$ with one eigenvector $v_3=v_4$ and a generalised eigenvector $v_g$. The solution will again asymptotically align with the eigenvector $v_4$ which has fourth component $k_3+\lambda _*^+<0$ and so the above proof by contradiction works in this case also. $\square$

Next we look at the case that the eigenvalue $\lambda _4$ is complex (i.e, $\epsilon _1^-<\epsilon <\epsilon _1^+$) and its real part is closest to zero.

### Lemma B.3

If the eigenvalue $\lambda _4$ is complex and its real part is larger than $\lambda _1$, hence closer to zero, then *y* generically converges to its steady state value $y=1$ in an oscillatory fashion, and so rebound occurs infinitely many times, but with exponentially decreasing magnitude.

### Proof

If the complex conjugate pair of eigenvalues $\lambda _{3,4}$ have real part that is closer to zero than $\lambda _1$, then the Hartman–Grobman Theorem (Crawford 1991) gives that generically trajectories will converge to the steady state in the phase space tangent to the (*y*, *w*) plane and will spiral around the steady state $(y,w)=(1,1)$. Each time the trajectory loops round the steady state, there will be a part of the trajectory with $y>1$ and so rebound occurs infinitely often, but with exponentially decreasing magnitude, which is determined by $\mathrm{Re}(\lambda _{3,4})= -(k_3+\epsilon )/2$.

As in the previous case, we also need to consider the possibility that for particular parameter values, the trajectory might approach tangent to a different eigenvector. We again note that the eigenvector $v_2$ points out of the phase space, and so a trajectory cannot approach the steady state tangent to this eigenvector. However, the eigenvector $v_1$ points into the phase space, since the first and second components of the eigenvector are both positive. Thus, we are unable to exclude the possibility that an orbit could approach the steady state tangent to this eigenvector for particular parameter values. However, this is very unlikely to occur in practice and is not a generic case. $\square$

### Proof of Lemma 5.13

This follows immediately by combining Lemmas B.1–B.3 and using that $\epsilon _1^-<\epsilon <\epsilon _1^+$ implies that $\lambda _4$ is complex. $\square$

Next we will consider the case that $\lambda _1$ is the eigenvalue closest to zero. The eigenvector $v_1$ corresponding to the eigenvalue $\lambda _1$ is given in (59). The first two entries in this eigenvector are the same as we had previously for the case with no feedback, and since both of these quantities are positive (see Lemma 2.7 of Aston et al. 2014), we must take a positive multiple of $v_1$ to ensure that *x* and *z* are positive in the direction of this eigenvector. We now consider the signs of the third and fourth components of this vector, although we note that since the steady state values for *y* and *w* are non-zero, there is no requirement that these entries be positive. First we determine the sign of the denominator in the expressions for the third and fourth components of $v_1$.

### Lemma B.4

If $\lambda _1$ is the least negative eigenvalue of the matrix (58) then

$$\begin{aligned} (k_3+\lambda _1)(\epsilon +\lambda _1)+\epsilon k_3h_0>0. \end{aligned}$$

### Proof

The eigenvalues $\lambda _3$ and $\lambda _4$ are found from the bottom right $2\times 2$ matrix in (58) and hence are roots of the characteristic polynomial

$$\begin{aligned} p(\lambda )=(k_3+\lambda )(\epsilon +\lambda )+\epsilon k_3h_0. \end{aligned}$$

We note that the quantity of interest is given by $p(\lambda _1)$.

Now $p(0)=\epsilon k_3(1+h_0)>0$. If the eigenvalues $\lambda _3$ and $\lambda _4$ are real and negative (with $\lambda _3<\lambda _4$) then $p(\lambda )>0$ for $\lambda \in (\lambda _4,0]$. Since $\lambda _1$ is the eigenvalue closest to zero, it must lie in this range and so in this case $p(\lambda _1)>0$.

Alternatively, if the eigenvalues $\lambda _3$ and $\lambda _4$ are complex, then $p(\lambda )$ has no real roots and so $p(\lambda )>0$ for all $\lambda$, and in particular $p(\lambda _1)>0$.

Thus, in both cases, $p(\lambda _1)>0$ as required. $\square$

### Proof of Lemma 5.14

The ratio of the third and fourth components of $v_1$ (which we denote by $v_{1,3}$ and $v_{1,4}$ respectively) is given by

$$\begin{aligned} \frac{v_{1,3}}{v_{1,4}}=-\frac{\epsilon +\lambda _1}{\epsilon h_0}. \end{aligned}$$

Thus, if $\epsilon +\lambda _1<0$, then $v_{1,3}$ and $v_{1,4}$ have the same sign and the proof of rebound given in Lemma B.1 holds in this case also.

If $k_1>k_4$, then we note from Lemma 2.8 of Aston et al. (2014) that $k_4+\lambda _1<0$ and so, using the result of Lemma B.4, we then see that $v_{1,4}<0$ in this case. Since we are taking a positive multiple of the eigenvector, this implies that *w* must approach its steady state value from below and so, by Corollary 5.11, rebound must occur in this case also.

As in previous cases, we should again consider the possibility that a trajectory could approach the steady state tangent to a different eigenvector. As previously, the eigenvector $v_2$ again points out of the invariant region since the first two entries have opposite sign. However, it is not possible to determine the global invariant manifold that approaches the steady state tangent to the eigenvectors $v_3$ or $v_4$ and so we can only state a generic result. $\square$
